# Hemozoin Promotes Lung Inflammation via Host Epithelial Activation

**DOI:** 10.1128/mBio.02399-20

**Published:** 2021-02-09

**Authors:** Shivang S. Shah, David A. Fidock, Alice S. Prince

**Affiliations:** a Division of Infectious Diseases, Department of Pediatrics, College of Physicians and Surgeons, Columbia University, New York, New York, USA; b Division of Infectious Diseases, Department of Medicine, College of Physicians and Surgeons, Columbia University, New York, New York, USA; c Department of Microbiology and Immunology, College of Physicians and Surgeons, Columbia University, New York, New York, USA; d Department of Pharmacology, College of Physicians and Surgeons, Columbia University, New York, New York, USA; University of Pittsburgh School of Medicine

**Keywords:** hemozoin, lung inflammation, malaria

## Abstract

Respiratory distress in severe malaria is associated with high mortality, but its pathogenesis remains unclear. The malaria pigment hemozoin (HZ) is abundant in target organs of severe malaria, including the lungs, and is known to be a potent innate immune activator of phagocytes. We hypothesized that HZ might also stimulate lung epithelial activation and thereby potentiate lung inflammation. We show here that airway epithelium stimulated with HZ undergoes global transcriptional reprogramming and changes in cell surface protein expression that comprise an epithelial activation phenotype. Proinflammatory signaling is induced, and key cytoadherence molecules are upregulated, including several associated with severe malaria, such as CD36 and ICAM1. Epithelial and extracellular matrix remodeling pathways are transformed, including induction of key metalloproteases and modulation of epithelial junctions. The overall program induced by HZ serves to promote inflammation and neutrophil transmigration, and is recapitulated in a murine model of HZ-induced acute pneumonitis. Together, our data demonstrate a direct role for hemozoin in stimulating epithelial activation that could potentiate lung inflammation in malaria.

## Abstract (precis)

Respiratory distress (RD) is a complication of severe malaria associated with a particularly high risk for death in African children infected with the parasite Plasmodium falciparum. The pathophysiology underlying RD remains poorly understood, and the condition is managed supportively.

## INTRODUCTION

Severe malaria caused by Plasmodium falciparum comprises several overlapping clinical syndromes, including cerebral malaria, severe malarial anemia, and respiratory distress, the latter associated with significantly increased mortality risk in African children (1, 2). Respiratory distress in malaria is understudied and likely multifactorial, a complex interplay between systemic disease burden and intrinsic lung injury, and often manifests as fulminant disease in spite of adequate antimalarial therapy (3–5).

Severe malaria is characterized by microvascular sequestration of infected erythrocytes and accumulation of the malaria pigment hemozoin (HZ) in target organs such as brain, spleen, placenta, and lungs (6–11). HZ is a potent innate immune effector in macrophages via the NLRP3 inflammasome and is associated with release of endogenous pyrogens that are thought to mediate the periodic fever of malaria infection (12–18). Intrapulmonary accumulation of HZ is observed in malaria-associated acute lung injury (MA-ALI) in both rodents and humans (7, 19) and may contribute to the pathogenesis of respiratory distress in severe malaria, as has been described for heme in other forms of acute lung injury (20, 21).

While the role of HZ in innate immune potentiation in phagocytes has been well studied, its lung epithelial effects are less well characterized. To better understand the role that HZ might play in potentiating MA-ALI, we studied its direct interaction with airway epithelial cells both *in vitro* and in a mouse model of pneumonitis. We hypothesized that HZ could potentiate lung inflammation by triggering an epithelial stress response involving proinflammatory pathways and barrier remodeling that would promote acute inflammation. The data we present show that HZ directly induces global changes in epithelial gene expression networks that result in host epithelial activation. Key features of this program induced by HZ include increased expression of proinflammatory mediators, upregulation of key cell surface adhesion molecules, and extensive changes in epithelial/extracellular matrix-associated gene expression and function, which together are associated with increased neutrophil transmigration. The net effect of this program of lung epithelial activation induced by HZ mirrors observed patterns of acute lung injury in severe malaria and yields mechanistic insight into its underlying pathogenesis.

## RESULTS

### Hemozoin induces global transcriptional reprogramming in airway epithelium.

We used transcriptome sequencing (RNA-seq) to probe changes in gene expression induced by HZ in airway epithelial cells. Following 24 h stimulation with HZ in media at three different concentrations (0, 100, and 200 μg/ml), total RNA was extracted as input for paired-end sequencing on Illumina HiSeq, with a total of six samples sequenced from two independent experiments. Sequencing data from each of the six samples were used to model gene expression as a quantitative trait using a read count-based method as implemented in DESeq2 (22). We employed two different modeling approaches, one treating HZ as a categorical variable (present or absent) and the other treating HZ concentration as a continuous variable. The former approach is optimized for detecting expression changes that rapidly saturate, while the latter captures more fine-tuned, dose-dependent changes, with results from both modeling approaches largely congruous with one another (Fig. S1 in the supplemental material).

10.1128/mBio.02399-20.1 FIG S1 Quality control plots of RNA-seq categorical and linear modeling. (A) *P* value distribution for differential expression analyses obtained for categorical (light) and linear (dark) modeling approaches. (B) Quantile-quantile plot of *P* values from linear versus categorical modeling. (C) Venn diagram showing overlap of top hits (*P*_adj_ < 10^−9^) between categorical and linear models. Individual list of top hits from both models enumerated in Tables S1 and S2. Download FIG S1, PDF file, 0.3 MB. Copyright © 2021 Shah et al. 2021 Shah et al. This content is distributed under the terms of the Creative Commons Attribution 4.0 International license.

10.1128/mBio.02399-20.2 FIG S2 Quality control plots of RNA-seq read count correlation, MA, and dispersion plots. (A) Read count correlation per gene (transformed by regularized logarithm as per DESeq2) between biologic replicates for each of three different HZ concentrations (0, 100, and 200 μg/ml). (B) MA (top) and dispersion plots (bottom) for categorical (left) and linear (right) models as generated by DESeq2. For MA plots, differentially expressed genes at *P*_adj_ values of <0.001 are highlighted in red. For dispersion plots, colors indicate estimated (black) and calculated (blue) dispersion, respectively, and red curve demonstrates fitted model for dispersion as a function of mean normalized counts. Download FIG S2, JPG file, 0.1 MB. Copyright © 2021 Shah et al. 2021 Shah et al. This content is distributed under the terms of the Creative Commons Attribution 4.0 International license.

**FIG 1:**
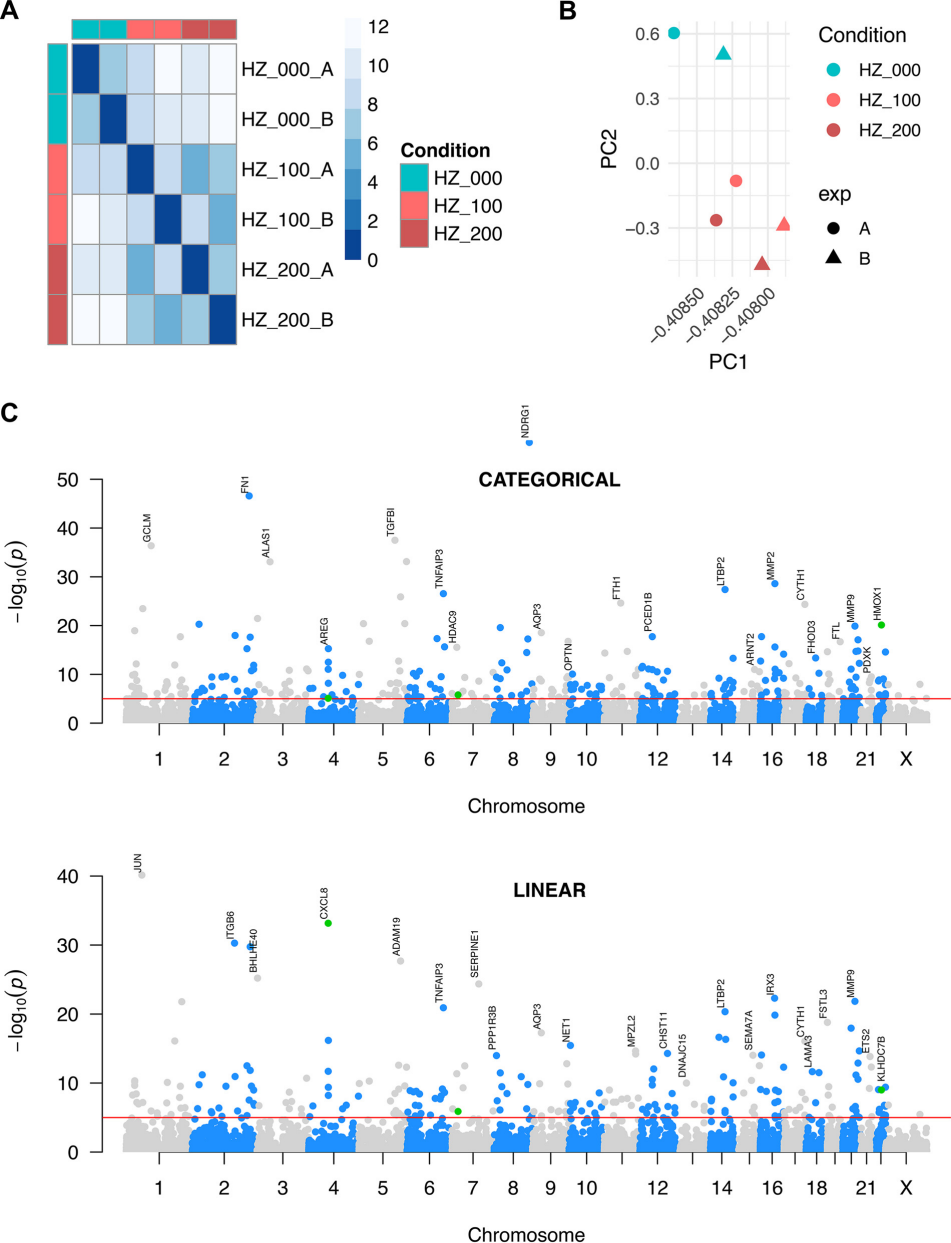
Global changes in the airway epithelial transcriptome are induced by HZ. Overview of transcriptomic data from HZ-stimulated human bronchial epithelial cells (16HBE), with two independent biologic replicates (circle, A; triangle, B) of three different experimental conditions (HZ = 0 μg/ml [cyan], 100 μg/ml [light red], or 200 μg/ml [dark red]). (A) Euclidean distance matrix of rlog-transformed (regularized logarithm transformation as per DESeq2) expression data per gene between samples. Darker shades indicate greater intersample relatedness. (B) Principal component analysis (PCA) plot of all six samples, using rlog-transformed gene-level expression data as input. (C) Whole-genome Manhattan plot of −log_10_-*P*_adj_ values (adjusted for multiple comparisons) for differential expression analyses according to categorical (top) and continuous (bottom) modeling approaches. Adjacent chromosomes are differentially colored; red line indicates *P*_adj_ value of 10^−5^, labeled points show top hit per chromosome with *P*_adj_ values of <10^−8^, and green points denote *CXCL8*/*IL-8* (chromosome 4), *IL-6* (chromosome 7), and *HMOX1* (chromosome 22).

**FIG 2:**
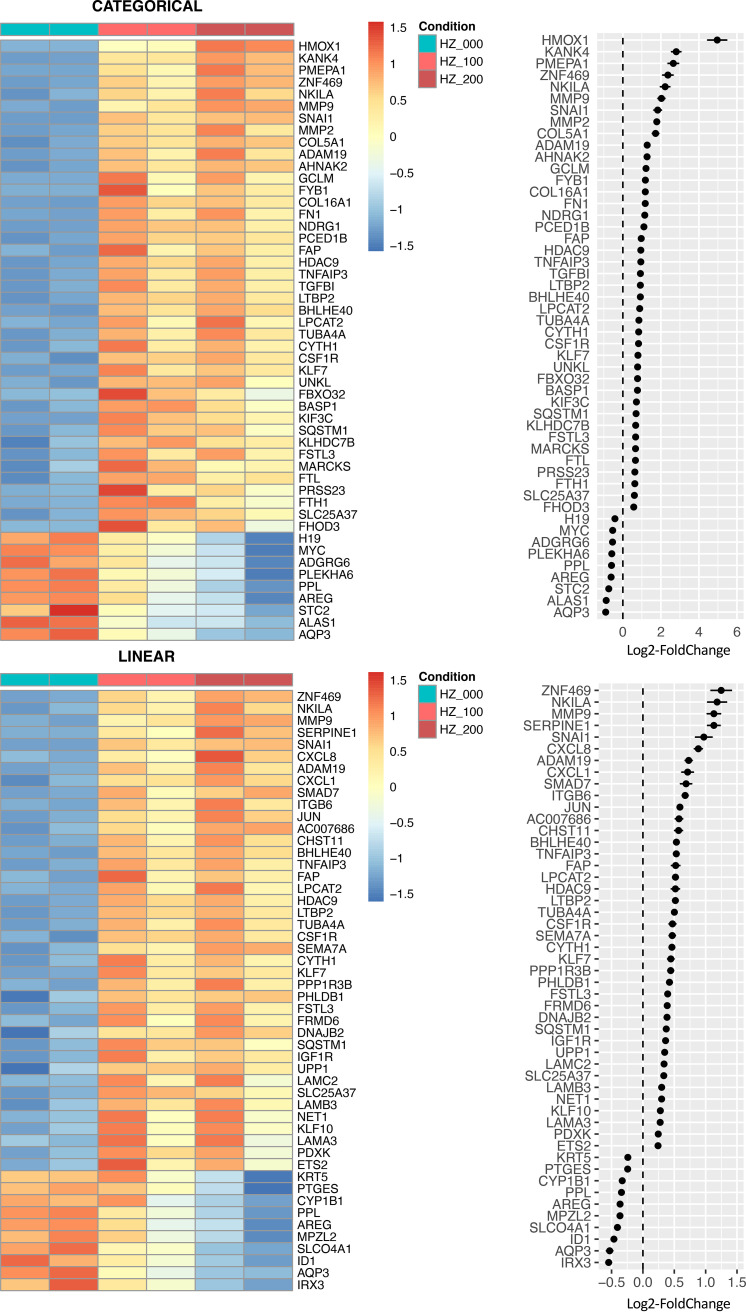
HZ induces differential expression of genes involved in epithelial activation. Summary of top hits from differential expression analyses of HZ-stimulated human bronchial epithelial cells (16HBE) under categorical (top) and linear (bottom) modeling approaches in DESeq2. The top 50 differentially expressed genes are shown (according to statistical confidence per *P*_adj_ rank), ordered by effect size. Heatmaps (left) show row-normalized expression values per sample (biologic replicates are adjacently paired) across three different experimental conditions (HZ = 0 μg/ml [cyan], 100 μg/ml [light red], or 200 μg/ml [dark red]). Dot plots (right) show direction and magnitude of expression change in response to HZ as log_2_-transformed fold change ± SEM. Genes displayed here were differentially expressed with high statistical confidence (*P*_adj_ < 10^−8^; see Tables S1 and S2 for details).

Global gene expression changes at the transcriptome-wide level were induced by HZ, as visualized by distance matrix heatmap and principal-component analysis (Fig. 1A and B). At the individual gene level, using both modeling approaches and after adjustment for multiple testing, we found over 500 signals of differential expression at an adjusted *P* value (*P*_adj_) of <10^−3^ across the genome. These included numerous strong signals that were suggestive of an overall epithelial activation phenotype (*P*_adj_ < 10^−9^; log_2_ fold change (log_2_FC) = [−0.9 to +5.0]) (Fig. 1C; Fig. 2; Tables S1 and S2).

10.1128/mBio.02399-20.7 TABLE S1 Top hits (categorical model). Download Table S1, PDF file, 0.10 MB. Copyright © 2021 Shah et al. 2021 Shah et al. This content is distributed under the terms of the Creative Commons Attribution 4.0 International license.

10.1128/mBio.02399-20.8 TABLE S2 Top hits (linear model). Download Table S2, PDF file, 0.10 MB. Copyright © 2021 Shah et al. 2021 Shah et al. This content is distributed under the terms of the Creative Commons Attribution 4.0 International license.

One major category of differentially expressed genes were those associated with epithelial and/or extracellular matrix remodeling. Among these were genes encoding numerous structural proteins (e.g., *FN1*, *COL5A1*, *COL16A1*, *KRT5*, *LAMA3*, *LAMB3*, *LAMC2*, and *TUBA4A*), junctional proteins (*CLDN1* and *PPL*), growth factor signaling mediators (*AREG*, *LTBP2*, *ITGB6*, *IGF1R*, and *SNAI1*), and metalloproteases (*ADAM19*, *FAP*, *MMP2*, and *MMP9*). Notably, among the latter group of metalloproteases, there are intriguing links to pulmonary pathophysiology in general, as well as target organ involvement in severe malaria. *ADAM19* (a disintegrin and metalloproteinase domain 19) has been previously linked to pulmonary function in genome-wide association studies (23), and MMP2 (matrix metallopeptidase 2) and MMP9 (matrix metallopeptidase 9) activity has been shown to potentiate CD8-positive (CD8^+^) T cell-mediated lung injury (24). Local MMP2 upregulation has been seen in experimental cerebral malaria (25), a clinical subphenotype of severe malaria also linked to CD8^+^ T cell-mediated tissue injury (10), and *MMP9* functional polymorphisms are thought to increase susceptibility to placental malaria (26).

Another major category of differentially expressed genes were proinflammatory mediators involved in cytokine signaling (*CXCL1*, *CXCL8*, *IL-6*, and *SEMA7A*), platelet activation (*SERPINE1*/*PAI1*, *LPCAT2*, *PTGES*, and *TGFBI*), tumor necrosis factor (TNF) and NF-κB signaling (*TNFAIP3*, *NKILA*, and *SQSTM1*), and oxidative stress response (*HMOX1* and *GCLM*). *CXCL1* (chemokine ligand 1) and *CXCL8/*interleukin 8 (*IL-8*) are canonical neutrophil chemotaxis factors associated with lung injury, and interleukin 6 (*IL-6*) is a master regulator of the acute phase response, associated with the acute respiratory distress syndrome and increased mortality in severe malaria (27, 28). *SEMA7A* (semaphorin 7A) participates in T cell-mediated inflammation and neutrophil guidance pathways associated with acute lung injury and is a known receptor for P. falciparum erythrocyte invasion (29–32). Platelet activation and aggregation have been associated with cytoadherence phenotypes involved in severe malaria (33, 34), and SERPINE1/PAI1 has been linked to various forms of lung injury (30, 35). Potent heme oxygenase induction here may reflect a compensatory response to oxidative stress, as has been described previously in experimental models of severe malaria and MA-ALI (36–38).

Several transcription factors (TFs) were among the genes differentially expressed (*P*_adj_ < 10^−3^; log_2_FC = [−0.5 to 1.2]), including *JUN* (upregulated) and *MYC* (downregulated), two canonical TFs with diverse functions that include response to cellular stress and cytokine signaling (Fig. S3). We also noted upregulation of TFs involved in collagen remodeling (*ZNF469*), epithelial-mesenchymal transition (*SNAI1*), regulation of cytokine production (*BHLHE40*), and response to oxidative stress (*KLF10*), mirroring the non-TF gene expression changes with respect to biologic processes involved.

10.1128/mBio.02399-20.3 FIG S3 Expression heatmap showing transcription factors. Differentially expressed transcription factors are shown, ordered by effect size, with heatmap showing row-normalized expression values per sample and dot plot showing direction and magnitude of expression change in response to HZ as log_2_-transformed fold change values ± SEM. Download FIG S3, PDF file, 0.05 MB. Copyright © 2021 Shah et al. 2021 Shah et al. This content is distributed under the terms of the Creative Commons Attribution 4.0 International license.

### Hemozoin alters expression networks involved in epithelial remodeling and proinflammatory signaling.

Our transcriptomic data highlighted a gene expression program suggestive of an epithelial activation phenotype, involving epithelial/extracellular matrix (ECM) remodeling and proinflammatory signaling pathways. To more precisely elucidate specific networks and pathways involved in the epithelial response to HZ, we used existing databases to examine protein-protein interaction networks and conduct gene set enrichment analyses.

**FIG 3:**
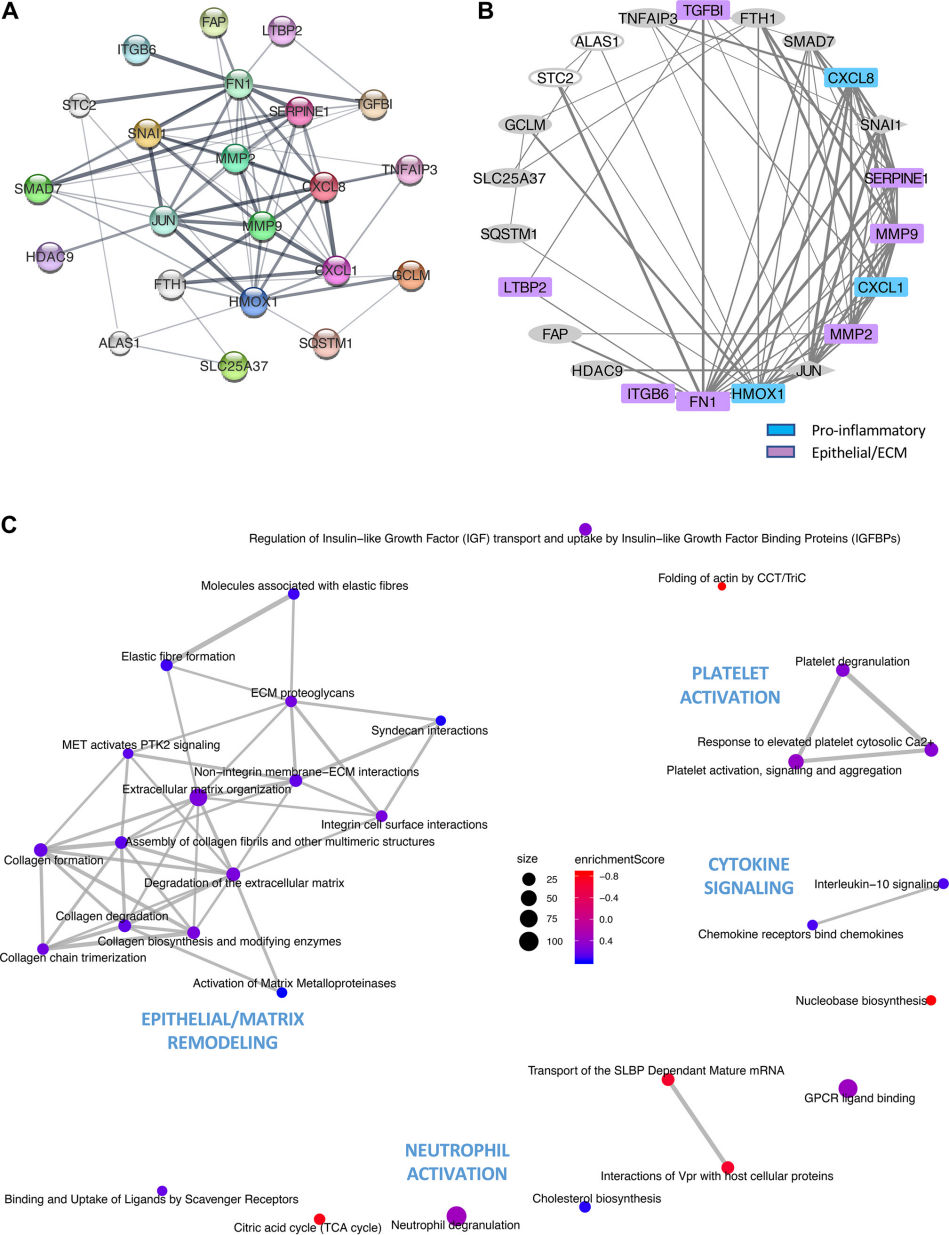
Networks of epithelial activation in response to HZ. Protein-protein interaction networks as annotated in StringDB for gene products of top hits from differential expression analyses of HZ-stimulated human bronchial epithelial cells (16HBE) are shown in top panels. Consensus list of top hits was used as input, comprising the union of top 20 hits according to *P*_adj_ values for categorical and linear models), with only nodes linked with moderate confidence per StringDB displayed (*n* = 22). (A) Force-directed, edge-weighted diagram where nodes are individual genes (arbitrary colors), with central nodes being those with the greatest number of and strongest evidence for protein-protein interactions and edge thickness scaled to StringDB interaction score. (B) Degree-sorted circular plot where nodes are individual genes colored according to GO function (blue, proinflammatory; magenta, epithelial/ECM; gray diamond, transcription factor) and direction of response to HZ (open, downregulated; filled, upregulated), with nodes sorted counterclockwise according to number of interactors, with edge thickness scaled to StringDB interaction score. (C) Network diagram depicting relationships between top pathway hits from gene set enrichment analyses (GSEA) probing Reactome pathways. Nodes are individual Reactome pathways, sized according to total number of constituent genes and colored according to GSEA enrichment score (blue, positive; red, negative). Edges connect nodes with shared genes, with line thickness correlated to number of shared genes between connected pathways.

A protein-protein interaction network of the top differentially expressed genes (per *P*_adj_ rank) in our data set was created using the StringDB database of known and predicted protein-protein interactions. This network, comprised of 22 interacting proteins with 69 known/predicted interactions, revealed strong interconnectivity between differentially expressed genes within the categories of proinflammatory (e.g., *IL-8* and *CXCL1*) and epithelial/ECM domains (e.g., *MMP2* and *MMP9*) (Fig. 3A and B), suggesting that proinflammatory and epithelial/ECM gene products were key mediators in the signaling induced by HZ.

To examine these gene-gene interactions further at the level of biologic pathways, we performed gene set enrichment analyses (GSEA) (39, 40), probing curated gene sets contained within the Molecular Signatures Database (MSigDB) collection, including KEGG, Gene Ontology Biologic Process (GO:BP), and Reactome. Among the main biologic pathways identified by GSEA, we observed particularly strong enrichment scores for those related to epithelial/ECM remodeling and proinflammatory signaling (Fig. S4 and S5). For example, top pathway hits included KEGG pathways such as “ECM-receptor interaction” (normalized enrichment score [NES] = 2.16; *P*_adj_ = 0.025) and “Cytokine-cytokine receptor interaction” (NES = 1.96; *P*_adj_ = 0.025) and Reactome pathways such as “Extracellular matrix organization” (NES = 2.26; *P*_adj_ = 0.012) and “Chemokine receptors bind chemokines” (NES = 1.97; *P*_adj_ = 0.012).

10.1128/mBio.02399-20.4 FIG S4 Gene set enrichment analyses (GSEA) sample plots. Selected standard enrichment plots from GSEA. Black vertical bars indicate individual genes in selected pathway, and green curve indicates running enrichment score (ES), where positive ES is associated with clustering of pathway genes among high-ranked genes (i.e., upregulated in response to HZ), and negative ES is associated with clustering among low-ranked genes in our data set (i.e., downregulated in response to HZ), where genes in our data set are ranked based on composite of effect magnitude and direction. Download FIG S4, PDF file, 0.09 MB. Copyright © 2021 Shah et al. 2021 Shah et al. This content is distributed under the terms of the Creative Commons Attribution 4.0 International license.

10.1128/mBio.02399-20.5 FIG S5 GSEA summary plots from KEGG, GO:BP, and Reactome. Shown are the top 30 pathway hits (*P*_adj_ < 0.05) for GSEA probing the KEGG (A), GO:BP (B), and Reactome (C) databases. Pathways are ranked by normalized enrichment score (NES), colored according to gene set size, with central table plot showing rank position of individual pathway genes across the ordered list of differentially expressed genes in our data set. Download FIG S5, PDF file, 0.2 MB. Copyright © 2021 Shah et al. 2021 Shah et al. This content is distributed under the terms of the Creative Commons Attribution 4.0 International license.

To further collate these individual pathway enrichment signals, we probed interpathway network relationships, visualizing top Reactome GSEA pathway hits via enrichment map network with ReactomePA (41). The network derived demonstrates prominent clustering within domains of epithelial and ECM remodeling, as well as cytokine/chemokine signaling and platelet activation (Fig. 3C), highlighting a concerted program of global epithelial activation via intercorrelated signaling networks.

### Hemozoin promotes an epithelial activation phenotype.

To further characterize the epithelial activation phenotype suggested by our transcriptomic data, we performed follow-on *in vitro* experiments to assess for functional consequences of HZ on lung epithelium, examining expression of key cell surface molecules and inflammatory mediators and characterizing junctional modulation.

**FIG 4:**
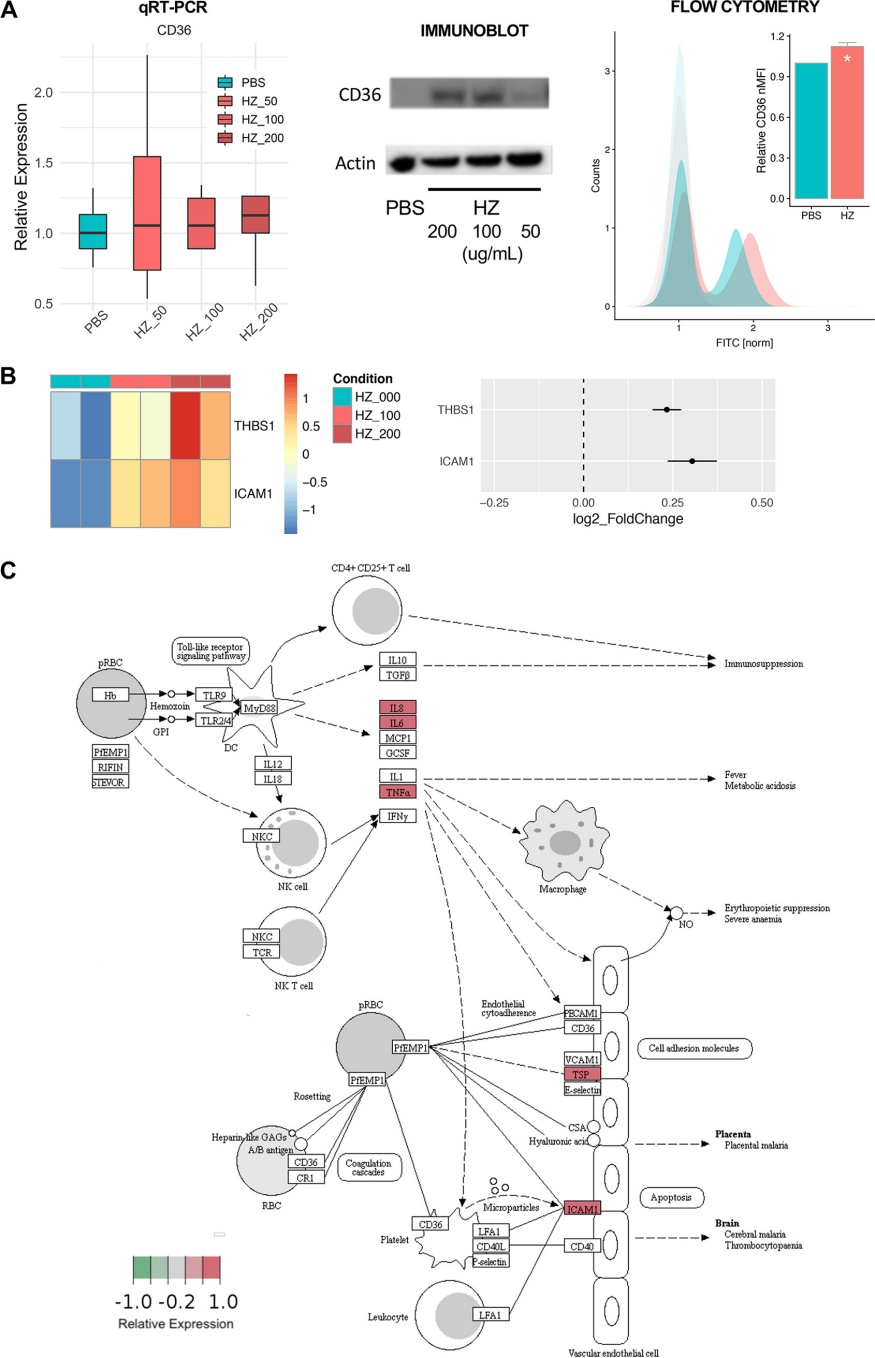
HZ induces expression of cell adhesion molecules important in malaria pathogenesis. (A) Posttranscriptional upregulation of CD36 in 16HBE cells in response to HZ. Panels show results of quantitative reverse transcription-quantitative PCR (RT-PCR) (left), immunoblot (middle), and flow cytometry (right). Colors indicate PBS (cyan) and HZ at 3 different concentrations (HZ = 50 μg/ml [light red], 100 μg/ml [medium red], or 200 μg/ml [dark red]). *, *P < *0.05; paired *t* test on normalized median fluorescence intensity (nMFI) relative to unstained control. Light-shaded curves represent isotype controls. Data representative of at least 3 independent experiments. Bar plot shows mean ± SEM; boxplot shows median with IQR. (B) HZ-induced transcriptional upregulation of *THBS1* (thrombospondin-1; *P_adj_* < 10^−7^; linear model) and *ICAM1* (intercellular adhesion molecule-1; *P_adj_* < 10^−4^; linear model) as per RNA-seq data. Heatmaps (left) show row-normalized expression values per sample, and dot plots (right) show direction and magnitude of expression change in response to HZ as log_2_-transformed fold change ± SEM. (C) KEGG pathway diagram of pathways known to be involved in the pathogenesis of malaria infection and severe malaria (KEGG ID hsa05144), with gene expression data from RNA-seq overlaid (relative expression as represented by the Wald statistic from differential expression analysis in DESeq2, which is proportional to log_2_-transformed fold change) colored according to increased (red) or decreased (green) expression in the presence of HZ.

Host cell surface molecules are important in severe malaria phenotypes, including respiratory distress, and several key host receptors have been identified. In particular, CD36 has been linked to cytoadherence phenotypes in severe malaria (33, 42–44) and malarial lung injury in particular, the latter via signaling through kinases involved cytoskeletal and junctional remodeling (45, 46). Given the strong transcriptomic network signal seen in epithelial/ECM remodeling pathways, we hypothesized that CD36 expression might also be affected. Interestingly, after stimulating airway epithelial cells with HZ for 24 h, we noted strong upregulation of CD36 at the protein level without affecting transcript abundance, suggesting a posttranscriptional mechanism of upregulation (Fig. 4A), as has been described for CD36 upregulation in other settings, e.g., hyperglycemia (47).

In our transcriptomic data, we also noted upregulation of two other malaria-associated cell adhesion molecules, thrombospondin-1 (*THBS1*) (*P*_adj_ < 10^−7^; log_2_FC = 0.23 ± 0.04; linear model) and intercellular adhesion molecule-1 (*ICAM1*) (*P*_adj_ < 10^−4^; log_2_FC = 0.30 ± 0.07; linear model) (Fig. 4B). THBS1 is a multifunctional cell surface molecule with roles in platelet activation and cell-matrix interactions via interaction with metalloproteases, integrins, and ECM proteins and has been associated with cytoadherence phenotypes in severe malaria (48). ICAM1 is another cytoadherence receptor relevant to severe malaria and MA-ALI (49–53) and is also critical for neutrophil trafficking and associated with other forms of acute lung injury (51, 54).

Taken as a whole, the overall program of epithelial activation induced by HZ, with upregulation of cytoadherence molecules and proinflammatory mediators, suggests that hemozoin itself may be a modifier in the host tissue response to severe malaria infection. Indeed, when examining an overlay of our RNA-seq expression data with the KEGG malaria pathogenesis diagram (KEGG ID hsa05144; Fig. 4C), we see that the expression phenotype stimulated by HZ in lung epithelium mirrors several known aspects of malaria pathogenesis more generally, which may have implications for nonimmune cell signaling by HZ more globally.

### Hemozoin induces junctional modulation and promotes neutrophil recruitment.

Our transcriptomic data suggested that the epithelial activation phenotype induced by HZ would be particularly functionally relevant to neutrophil recruitment, given the observed upregulation of *ICAM1* and network signals seen in epithelial matrix remodeling, chemokine signaling, and neutrophil activation domains in response to HZ (Fig. 3C). Neutrophils are important in diverse types of acute lung injury, including MA-ALI, as they generate local tissue damage through the elaboration of toxic proteases and release of reactive oxygen species (51, 54–58). We hypothesized that potent induction of neutrophil chemokines, upregulation of cell adhesion molecules, and changes in epithelial/ECM remodeling might also be associated with neutrophil recruitment to the airway, and we performed follow-on *in vitro* studies to further investigate.

**FIG 5:**
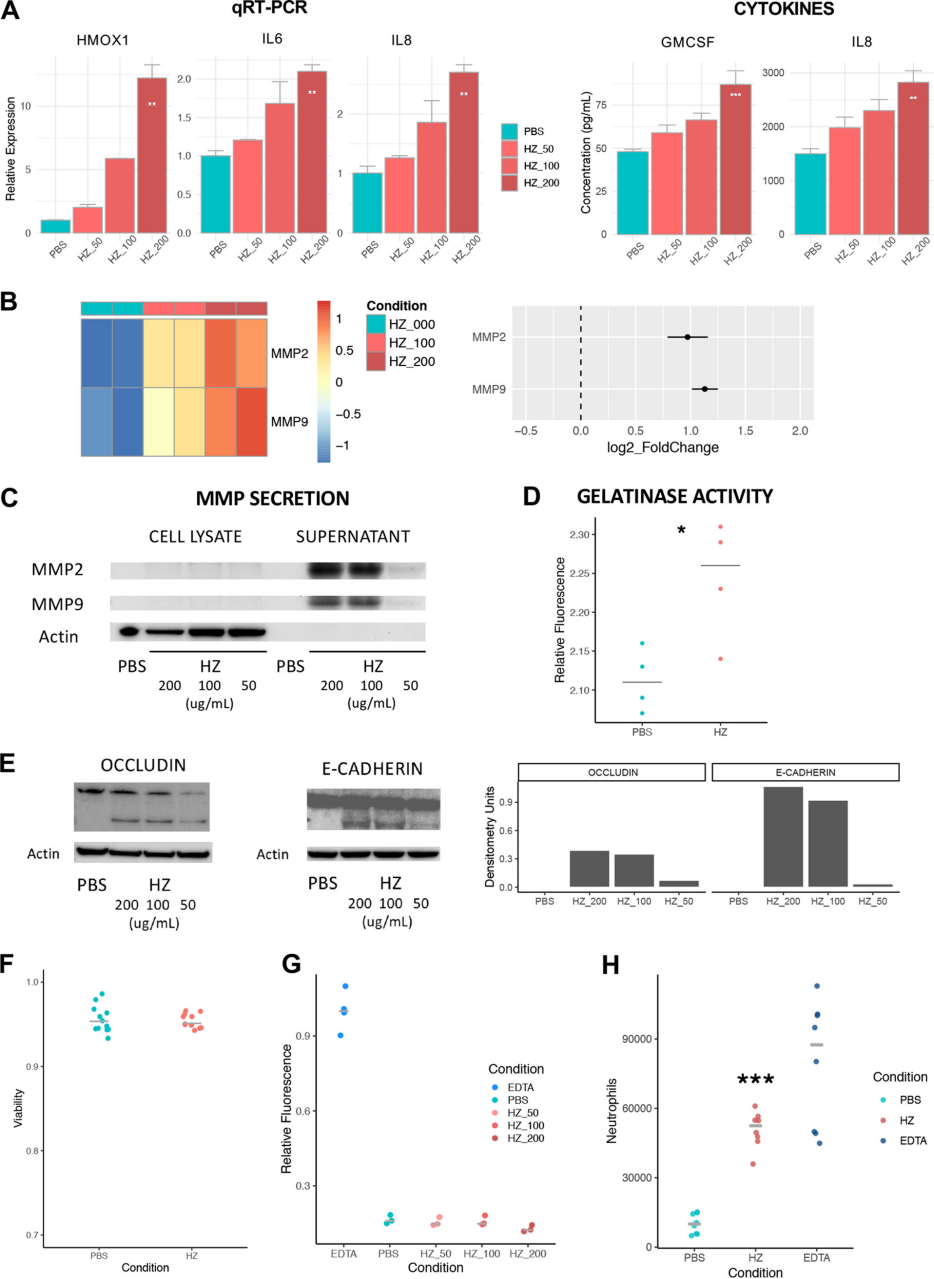
HZ induces junctional modulation and promotes neutrophil transmigration. (A) HZ induces dose-dependent upregulation of proinflammatory mediator expression, including *HMOX1*, *IL-6*, and *IL-8* (left, quantitative RT-PCR) and neutrophil chemokines GM-CSF and IL-8 (right, ELISA). **, *P < *0.01; ***, *P < *0.001; linear regression. Data representative of at least 2 independent experiments. Bar plots depict mean ± SEM. Colors indicate treatment with PBS (cyan) or HZ (red; HZ = 50, 100, or 200 μg/ml). (B) HZ-induced transcriptional upregulation of *MMP2* (matrix metallopeptidase 2; *P*_adj_ < 10^−6^; linear model) and *MMP9* (matrix metallopeptidase 9; *P_adj_* < 10^−19^; linear model) in airway epithelium (16HBE) as per RNA-seq data. Heatmaps (left) show row-normalized expression values per sample, and dot plots (right) show direction and magnitude of expression change in response to HZ as log_2_-transformed fold change ± SEM. (C) Secretion of MMP2 and MMP9 by 16HBE epithelial cells as shown by immunoblot of cell culture supernatant. Data representative of at least 2 independent experiments. (D) Gelatinase (MMP2/MMP9) activity by cellular fluorescence assay in 16HBE cells stimulated with PBS or HZ (200 μg/ml). Fluorescence intensity relative to unlabeled control shown. *, *P < *0.05; *t* test. Data representative of at least 2 independent experiments. (E) Generation of cleavage products of occludin (left) and E-cadherin (right) in response to HZ, as shown by immunoblot, alongside densitometry graph for cleaved fragment (relative to actin, arbitrary units). Data representative of at least 2 independent experiments. (F) 16HBE cell viability after 24 h of exposure to PBS or HZ (200 μg/ml) as measured by Trypan blue exclusion. No significant differences were seen between PBS (cyan) and HZ (red). Data pooled from 3 independent experiments. (G) Permeability of FITC-dextran across air-liquid interface Transwell cultures of 16HBE cells. Permeability expressed as fluorescence relative to EDTA-treated cells (blue, positive control). No significant differences were seen between PBS- and HZ-treated samples. Data representative of 3 independent experiments. (H) Transmigration of human neutrophils across polarized air-liquid interface Transwell cultures of 16HBE cells treated with PBS (cyan), HZ (red), or EDTA (blue, positive control). Data pooled from three independent experiments. ***, *P < *0.001; Kruskal-Wallis rank sum test.

We noted potent induction of several inflammatory mediators in lung epithelium stimulated with HZ, including several neutrophil chemokines (Fig. 5A). This included dose-dependent expression changes in *IL-6* and *CXCL8*/*IL-8* transcripts, which were complemented by direct cytokine measurement showing increased secretion of IL-8 and granulocyte-macrophage colony-stimulating factor (GM-CSF). The latter represent two key chemokines involved in neutrophil recruitment and activation, and known to be elevated in mouse models of MA-ALI (19, 46, 59, 60).

Our transcriptomic data showed strong upregulation of *MMP2* and *MMP9*, two matrix metallopeptidases associated with junctional and ECM remodeling (Fig. 5B; *MMP2*, *P*_adj_ < 10^−6^, log_2_FC = 0.97 ± 0.18, linear model; *MMP9*, *P*_adj_ < 10^−19^, log_2_FC = 1.13 ± 0.12, linear model), and our follow-on *in vitro* experiments confirmed this, demonstrating increased secretion and enzyme activity of MMP2 and MMP9 (Fig. 5C and D). Junctional protein cleavage by MMPs is known to be important in junctional modulation that facilitates neutrophil transmigration. In prior work from our group, we have shown that Pseudomonas aeruginosa is able to induce proteolytic cleavage of junctional proteins occludin and E-cadherin, which allows for epithelial junction modulation without overtly breaching barrier integrity and facilitates neutrophil transmigration across airway epithelia (61). As occludin and E-cadherin are substrates for MMP2 and MMP9 (62, 63), we speculated that HZ might induce a similar phenotype. Upon stimulation of airway epithelium with HZ, we noted increased production of occludin and E-cadherin cleavage products (Fig. 5E). This junctional modulation, in concert with the cytokine gradient induced by HZ, was associated with increased neutrophil transmigration across an epithelial monolayer in Transwell culture, with no effect on viability or overall permeability to dextran (Fig. 5F, G, and H), similar to the junctional modulation phenotype seen with bacterial stimuli (61).

### Hemozoin induces airway pneumonitis *in vivo*.

**FIG 6:**
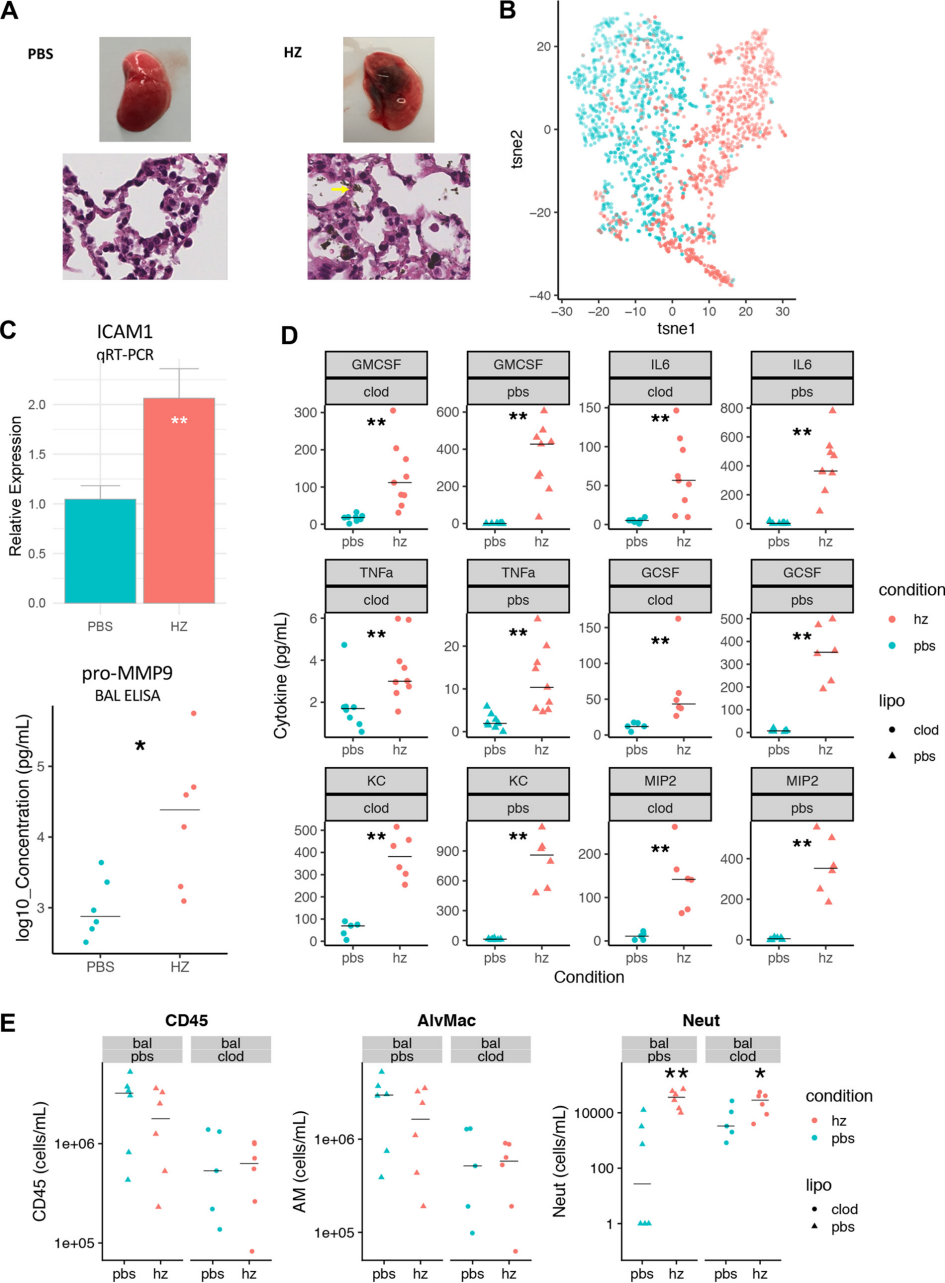
HZ induces airway pneumonitis *in vivo*. (A) Gross and microscopic pathology of lungs after intranasal PBS (left) or HZ (right) administration, confirming diffuse distribution of HZ particles (yellow arrow). (B) Two-dimensional t-SNE plot of CD45^+^ immune cells in bronchoalveolar lavage (BAL) fluid obtained from mice 6 h after intranasal PBS (cyan) or HZ (red) administration as visualized by t-SNE algorithm, using CD45^+^ DAPI^−^ as input cell population and measured parameters of FSC, SSC, Ly6G, CD11b, SiglecF, MHC-II, CD31, CD36, and EpCAM. Two-dimensional t-SNE map shown; units are arbitrary; points shaded according to relative Ly6G expression. Data representative of 2 independent experiments. (C) Levels of expression of ICAM1 (quantitative RT-PCR [qRT-PCR]) and pro-MMP9 (enzyme-linked immunosorbent assay [ELISA]) in lung following PBS (cyan) or HZ (red) administration. Data pooled from at least 2 independent experiments. *, *P < *0.05; **, *P < *0.01; Wilcoxon rank sum test. Bar plot depicts mean ± SEM. (D) Cytokines in BAL fluid obtained from mice 6 h after intranasal PBS (cyan) or HZ (red) administration, following pretreatment with either PBS- (triangle) or clodronate-loaded (circle) liposomes. Data pooled from at least 2 independent experiments. All differences between PBS and HZ groups were significant; *P < *0.01 by two-way ANOVA. (E) Immune cells in BAL fluid obtained from mice 6 h after intranasal PBS (cyan) or HZ (red) administration, following pretreatment with either PBS- (triangle) or clodronate-loaded (circle) liposomes. Data pooled from 2 independent experiments. *, *P < *0.05; **, *P < *0.01; Wilcoxon rank sum test.

We next examined the effect of hemozoin on airway epithelium *in vivo* in a mouse model of hemozoin-induced acute pneumonitis, employing airway administration of HZ to focus specifically on the acute airway epithelial response. Given that the most abundant immune cell population in the airway is the macrophage (>95% of all cells) and that HZ exerts a potent immunostimulatory effect on macrophages (13), we also sought to establish if induction of pulmonary inflammation was macrophage independent by clodronate depletion. Mice were challenged with either intranasal phosphate-buffered saline (PBS) or HZ (50 μg HZ = 50 μl of 1 μg/μl HZ in PBS) and sacrificed 6 h later in order to assess the acute inflammatory response to HZ (Fig. 6A). Bronchoalveolar lavage (BAL) fluid was analyzed for cytokine levels, as well as immune cell populations by multicolor flow cytometry. In our clodronate experiments, intranasal pretreatment with liposomes loaded with either PBS or clodronate (50 μl at 5 mg/ml) was performed 24 h prior to HZ challenge according to an established airway macrophage (AM) depletion protocol (64), yielding approximately 90% depletion of AMs (Fig. 6E).

In this *in vivo* work, we noted a similar pattern of changes induced by HZ as seen *in vitro*, with induction of proinflammatory cytokines, including tumor necrosis factor alpha (TNF-α) and neutrophil chemokines granulocyte colony-stimulating factor (G-CSF), GM-CSF, IL-6, KC, and MIP2, as well as increased expression of pro-MMP9 and key neutrophil adhesion receptor ICAM1 (Fig. 6C and D). In examining cell populations, we noted an acute ingress of neutrophils and an overall change in the overall immune cell marker phenotype as visualized by the *t*-distributed stochastic neighbor-embedding (t-SNE) algorithm (Fig. 6B and E). Even in the setting of AM depletion, HZ was still able to induce production of proinflammatory cytokines (G-CSF, GM-CSF, IL-6, KC, MIP2, and TNF-α) and induce neutrophil recruitment (Fig. 6D and E), similar to what was observed in our *in vitro* experiments. Notably, the overall pattern of airway epithelial response to HZ, in terms of specific cytokine induction and neutrophil recruitment, is similar to that previously seen in rodent malaria models of MA-ALI (19, 59, 60).

## DISCUSSION

Respiratory distress (RD) is a major, frequently fatal complication of severe malaria, often associated with intrinsic lung injury along the spectrum of ALI/ARDS (acute lung injury/acute respiratory distress syndrome). RD exhibits clinical heterogeneity at the level of both host and parasite, affecting children and adults alike across a wide geographic range, with several *Plasmodium* species implicated to date, and often developing in spite of timely antimalarial therapy and clearance of peripheral blood parasitemia (3). The known persistence of HZ in target organs of severe malaria (6, 19), coupled with pathology findings of endothelial dysfunction and diffuse alveolar damage in humans with MA-ALI (3, 65), led us to hypothesize that an ongoing epithelial inflammatory response to HZ might potentiate lung inflammation through direct effects on airway epithelial cells, similar to what has previously been described for heme/hemoglobin-mediated ALI (20, 21). Through a combination of transcriptomic and bioinformatic approaches, *in vitro* functional studies, and an *in vivo* model of acute pneumonitis, we show that HZ directly induces a diverse program of epithelial activation and potentiates lung inflammation. This epithelial activation response, which involves induction of proinflammatory stress response mediators (CXCL1, IL-6, IL-8, GM-CSF, and HMOX1), increased expression of cell surface markers relevant to cytoadherence and severe malaria (CD36, ICAM1, and THBS1), and epithelial barrier remodeling, is associated with neutrophil recruitment into the airway.

The program of epithelial activation induced by HZ shown in our data complements prior findings from rodent models of severe malaria and lung injury. One compelling aspect of the simplicity of our experimental model lies in how synthetic HZ, independent of other bioactive adherent molecules from either parasite or host, is able to replicate some of the molecular phenotypes implicated in malaria pathogenesis (KEGG ID hsa05144; Fig. 4).

For example, prior work with MA-ALI has demonstrated a key role for neutrophil recruitment in the pathogenesis of lung injury. Neutrophil infiltration into the airway has been seen in commonly used Plasmodium berghei models of MA-ALI, associated with induction of neutrophil chemokines such as KC/CXCL1 and IL-6 (19, 59, 60) and correlated with pulmonary HZ burden (19). Treatment with antineutrophil antibodies early in the course of P. berghei ANKA infection has even been found to abrogate pulmonary disease and decrease mortality (55). Our data show how the program of epithelial activation induced by HZ is associated with neutrophil recruitment and thus may potentiate lung inflammation. One aspect of this effect is the elaboration of proinflammatory neutrophil chemokines that we observed *in vitro* and *in vivo* in response to HZ, including IL-6, IL-8, CXCL1, and GM-CSF. Notably, in our airway model, this induction of neutrophil chemokines and neutrophil infiltration occurred even with depletion of the dominant population of airway immune cell, the airway macrophage, which is known to be potently activated by HZ via the NLRP3 inflammasome (13, 15). Apart from cytokine induction, HZ also induced upregulation of ICAM1, a cell adhesion molecule integral to neutrophil trafficking and also implicated in various forms of acute lung injury (51, 54) in addition to its well-known role in severe malaria cytoadherence phenotypes (49–53).

Perhaps the most profound impact of HZ on lung epithelial activation was changes induced in epithelial/ECM remodeling pathways. Prior work has noted the importance of CD36 and Src family kinase signaling to endothelial barrier dysfunction and development of MA-ALI (45, 66). Parasite sonicates are known to induce endothelial barrier dysfunction in an Src family kinase-dependent manner (66), and CD36 signaling through the Fyn kinase (another member of the Src kinase family) pathway is critical to endothelial barrier dysfunction and development of MA-ALI in a P. berghei ANKA model. Our data suggest that HZ itself, independent of other parasite factors, can play a similar role in epithelial junctional remodeling, altering this axis by upregulating CD36 expression and modulating the expression of key aspects of the downstream cytoskeletal/junctional morphology pathways through which these tyrosine kinases signal. Past studies have also linked induction of MMP2 and MMP9, and degradation of the ECM, to immune-mediated acute lung injury (24, 56), and local MMP2 upregulation has been observed in experimental cerebral malaria (25). MMPs are also known to modulate barrier function via junctional protein cleavage, and here we found that MMP2 and MMP9 were significantly upregulated in response to HZ, coinciding with increased proteolytic breakdown of two of their known substrates, occludin and E-cadherin (62, 63). Similar to what has been observed for heat-killed Pseudomonas aeruginosa (61), this modulation of junctional barrier function, while not affecting the gross integrity of the epithelial barrier (i.e., macromolecule permeability), was associated with increased neutrophil egress in conjunction with proinflammatory cytokine upregulation.

One consequence of neutrophil infiltration into the lungs in ALI relates to release of reactive oxygen species and reactive nitrogen species (ROS and RNS, respectively), which have been associated with lipopolysaccharide (LPS)- and malaria-associated ALI (45, 58, 67). In our data, we noted especially strong induction of *HMOX1*, presumably a compensatory response to the oxidant stress elicited by HZ (12, 14). Induction of *HMOX1* in response to carbon monoxide (CO) has been shown to protect against MA-ALI in murine models (37, 38, 68, 69). Given the persistence of HZ in target organs after natural malaria infection (19), the degree of local *HMOX1* induction in the lung may yield insight into differential susceptibility to MA-ALI in different individuals exposed to repeated infections in endemic settings. Given the relative hypoxemia associated with microvascular sequestration of infected erythrocytes in the lung and the known contribution of ROS to lung injury in hypoxemic states (70), ROS generated in response to HZ likely augments lung injury in the setting of endovascular sequestration and paracellular leak in a manner analogous to that described for heme in other forms of lung injury (20, 21). One could envision a feedforward loop of local epithelial and endothelial activation, involving cytoadherent infected erythrocytes and locally concentrated hemozoin released from HZ-rich trophozoites, the latter further stimulating factors that increase sequestration and further epithelial and endothelial activation, thus exacerbating lung injury. Our focus here was on airway epithelial responses, but another area for future investigation would be lung endothelial activation, especially in relation to cell adhesion molecule upregulation and cytoadherence phenotypes that have been linked with severe malaria.

Our work is unique within the HZ literature in its focus on the lung epithelium, as opposed to the professional immune cell. While HZ preparations are variable in their morphology and adherent biomolecules (e.g., nucleic acids, protein, and lipids) based on source and means of preparation, synthetic HZ is identical to natural hemozoin with respect to chemical, spectroscopic, and crystallographic characteristics (71). We chose a synthetic HZ preparation to avoid the confounding effect of other adherent costimulant biomolecules that can differentially precipitate with HZ according to preparation method when using biological samples, as this may have contributed to past controversies regarding HZ and innate immune signal transduction biology (72). We chose physiologic concentrations of HZ that have been estimated to be released with each cycle of schizont rupture in natural infection (73), e.g., if a 10-kg child had 5% parasitemia, an estimated 1 g HZ released into a blood volume of 0.8 liters per cycle would equate to approximately 1.2 mg/ml (approximately 1.2 mM heme equivalents). The concentrations of HZ used are similar to those used in prior *in vitro* studies of HZ and inflammasome activation (12, 13), and our intranasal load of HZ delivery (50 μg = 50 nmol) was similar to the pulmonary levels of HZ observed after intravenous (i.v.) administration in mice by Deroost et al. (19).

We focused here on the response in the airway epithelium, which differs from the alveolar epithelium with respect to physiologic and immune function (74). The alveolar epithelium, together with microvascular endothelium, constitutes the critical initial interface relevant to barrier dysfunction in lung injury, especially with respect to hematogenous triggers such as HZ. However, as single-cell technologies have recently elucidated, there is both subtle heterogeneity and remarkable fluidity across lung epithelial subtypes, across both airway and alveolus, and interestingly, transcriptional responses across various types of lung epithelia tend to globally coaggregate in these studies (75, 76). Thus, given its critical role in the innate inflammatory response and barrier regulation, we would expect the alveolar epithelium to retain many key features of the airway epithelial activation response that was seen. Additionally, it is notable that both our *in vitro* and *in vivo* results comport with one another, as the latter likely reflects contributions from both types of epithelium, given the diffuse distribution of HZ down to the level of the airspace seen on histopathology (Fig. 6A). Nonetheless, further work is needed to understand the alveolar epithelial response in its physiologic context, e.g., lung-on-a-chip or organoid culture, or from *ex vivo* isolated epithelial cells using single-cell RNA-seq.

We employed a simple proof-of-concept *in vivo* model to focus primarily on the acute response in lung epithelium. To that end, we chose an intranasal delivery method, as systemic HZ delivery would invoke a potent systemic inflammatory response. Interestingly, the specific pattern of lung inflammation observed here largely comports with that seen previously with systemic HZ (19). We also chose a short-term time point (6 h) in order to focus on the acute inflammatory response in the airway. While the acute inflammatory response observed *in vivo* mirrored responses seen *in vitro* at a longer time point (24 h), a closer study of the temporal course of epithelial activation *in vivo* would be informative.

Given the pleiotropy of the epithelial activation response induced by HZ, there are limitations with respect to causal inference in this study, and several key questions remain. Further investigation should address whether there is a key central effector that could be a target for inhibition, e.g., MMPs or CD36/Fyn signaling. Likewise, it will also be important to identify the proximal sensors for HZ on lung epithelium and whether they localize to the cell membrane or endocytic vesicles. CD36, a multifunctional scavenger receptor key in endocytosis of other biocrystalline substances, might conceivably play a role (77).

Malaria-associated lung injury is a complex phenomenon, with many factors to consider, including parasite species and cytoadherence virulence factors, complex host genetics and immunity, local endemicity patterns, and comorbidities including malnutrition and anemia. While our simplified model does not capture the complexity of natural infection, it does provide proof of principle for how innate immune mediators such as HZ can act directly on nonimmune cells such as lung epithelium to modulate the local host response to infection and potentiate local tissue inflammation. The diverse pathways affected provide numerous targets for further functional characterization and potential therapeutic intervention in malarial lung injury.

## MATERIALS AND METHODS

### Cell culture.

16HBE human bronchial epithelial cells (courtesy of D. Gruenert, California Pacific Medical Center Research Institute) were grown in BronchiaLife (Lifeline) media supplemented with growth factors, 1% penicillin/streptomycin, and 5% fetal bovine serum. Routine surveillance testing was performed to assess for *Mycoplasma* contamination.

### Hemozoin.

Synthetic HZ (Invivogen) was prepared by acidic method (78) and certified endotoxin free. HZ stock solution was prepared at 5 mg/ml HZ in PBS, sonicated and vortexed prior to use, and used within 1 month of initial preparation.

### *In vitro* studies.

Cells were seeded onto 24-well plates and grown to confluence and then treated with either PBS in media or HZ in media at either 50, 100, or 200 μg/ml concentration for 24 h. The 24-h time point was chosen based on pilot studies showing more stable, pronounced expression differences in genes of interest than the 4-h time point and also after prior *in vitro* work done by others (12). Plates were then washed with PBS and cells lysed in either radioimmunoprecipitation assay (RIPA) plus HALT buffer for immunoblotting (Thermo Scientific; RIPA, 20 mM Tris-HCl, 50 mM NaCl, 0.1% SDS, 1% Triton X-100, 10% glycerol, 2 mM EDTA, and 0.5% sodium deoxycholate; HALT, protease and phosphatase inhibitor), or TRK buffer for RNA extraction (Omega Bio-Tek). Gelatinase (MMP2/MMP9) activity was quantified using a commercial kit (Calbiochem; catalog no. CBA003), which uses a specific quenched fluorescent collagen substrate specific for MMP2/MMP9. Cell cultures were incubated with substrate, 1 mM *p*-aminophenylmercuric acetate (APMA) (activator) in cell culture media with either PBS or HZ for 24 h, and then detached and washed three times prior to reading cellular fluorescence on a flow cytometer. Fluorescence intensity relative to unlabeled (substrate-free) control sample was recorded for each experimental condition.

### Transwell permeability and neutrophil transmigration experiments.

Previously established methods were used (61, 79). In brief, 16HBE cells were seeded in inverted fashion onto 3.0-μm-diameter Transwell permeable supports (Costar 3462; Corning) coated with collagen (PureCol; Advanced BioMatrix) within 12-well plates. Cells were first allowed to adhere to inverted Transwells for 8 h and then reoriented in normal configuration and fed with media on both apical and basolateral surfaces for at least 3 days until confluent. At that point, epithelial monolayers were allowed to polarize by establishing air-liquid interface conditions. This was achieved by removing media from the bottom main chamber (apical side) and only feeding media into a smaller Transwell chamber (basolateral side), with air-liquid interface conditions maintained for at least 5 days prior to assays. The integrity of monolayers established is shown in dextran permeability assays (Fig. 5C). Cell viability after 24 h treatment with PBS or HZ (200 μg/ml) was assessed by Trypan blue exclusion following cell detachment. Monolayers were treated on the apical surface with either PBS or HZ at indicated concentrations for 24 h prior to assessing permeability or transmigration. EDTA (25 mM) was used as a positive control for both permeability and migration experiments. For dextran permeability assays, basolateral treatment with 1 mg/ml fluorescein isothiocyanate (FITC)-labeled dextran 3,000 molecular weight (MW) (catalog no. D3305; Molecular Probes) was applied for 30 min at 37°C with fluorescence in the bottom chamber and then assayed using a plate fluorometer (Tecan). For neutrophil transmigration experiments, human neutrophils from healthy volunteers (Institutional Review Board [IRB] protocol AAAR1395) were isolated using standard techniques (79) of sequential gelatin sedimentation, followed by hypotonic lysis of erythrocytes, and, lastly, density gradient centrifugation (Histopaque-1077; Sigma). Neutrophils were quantified on a hemocytometer using Trypan blue exclusion, which differentiates live neutrophils from any dead, sloughed epithelial cells. Neutrophil purity was verified using flow cytometry for CD66b (data not shown). Approximately 10^5^ neutrophils were loaded per Transwell on the basolateral side, allowed to migrate for 2 h, and then counted in an apical chamber using a hemocytometer.

### Mice.

All animal use protocols were devised in accordance with ethics guidelines and institutional requirements of Columbia University Medical Center and approved by its Institutional Animal Care and Use Committee under protocol AC-AAAR1401. C57BL/6 female mice were obtained from Jackson Laboratories (Bar Harbor, ME), housed in a pathogen-free environment, and were 7 to 8 weeks old at the time of experiments.

### Hemozoin pneumonitis.

Mice were treated with either 50 μl PBS or HZ (1 μg/μl HZ in PBS = 50 μg HZ) delivered via intranasal administration and then sacrificed after 6 h. For clodronate depletion experiments, liposomal pretreatment was performed 24 h prior, where mice were pretreated with either 50 μl of liposomal PBS or liposomal clodronate at 5 mg/ml (clodronateliposomes.org). All intranasal treatments were done under procedural sedation with 100 mg/kg ketamine plus 5 mg/kg xylazine (Henry Schein). Euthanasia was achieved using pentobarbital/phenytoin (Euthasol; Virbac). After euthanasia, airway cells were isolated via bronchoalveolar lavage (BAL) fluid using three sequential 1-ml washes of sterile PBS, then recovered by centrifugation (initial supernatant reserved for cytokine and protein quantitation), resuspended in RBC lysis buffer, and then washed twice in fluorescence-activated cell sorting (FACS) buffer (10% fetal bovine serum and 0.1% sodium azide in PBS) prior to downstream use. Aliquots of BAL fluid and lung homogenate were reserved in RIPA plus HALT and TRK buffers for immunoblotting and RNA extraction, respectively, with the remainder used for flow cytometry as described below.

### Immunoblotting.

Whole-cell lysates stored in RIPA plus HALT were quantified via colorimetry (Precision Red assay; Cytoskeleton Inc.), and then equimolar amounts of lysate were loaded onto Bolt 10% Bis-Tris for electrophoretic separation. Proteins were transferred from gel to membrane via iBlot dry blotting system (Thermo Fisher), and membranes were blocked for 1 h in 5% milk in TBST (Tris-buffered saline with Tween 20; 50 mM Tris, pH 7.5, 150 mM NaCl, and 0.05% Tween 20). Labeling with primary antibody was done at 4°C overnight at 1:200 dilution, while probing with secondary antibody was done at 25°C for 1 h at 1:10,000 dilution. Primary antibodies used included anti-occludin (catalog no. sc-133256; Santa Cruz Biotechnology), anti-E-cadherin (catalog no. ab1416; Abcam), anti-MMP9 (catalog no. sc-21733; Santa Cruz Biotechnology), anti-MMP2 (catalog no. sc-13595; Santa Cruz Biotechnology), anti-CD36 (catalog no. sc-7309; Santa Cruz Biotechnology), and anti-beta-actin (catalog no. A5441; Sigma). The secondary antibody conjugated to horseradish peroxidase (HRP) used was anti-mouse-IgGκ-HRP (catalog no. sc-516102; Santa Cruz Biotechnology).

### Flow cytometry.

For *in vitro* work, cells were detached from tissue culture plates enzymatically using TrypLE Express (Thermo Fisher) and then washed in media and recovered by centrifugation. Cells were then fixed in 2% paraformaldehyde in PBS and permeabilized with 0.05% saponin in FACS buffer prior to staining. The primary antibody used was anti-CD36 (catalog no.sc-7309; Santa Cruz Biotechnology) at 1:100 dilution, and the secondary antibody used was Alexa Fluor 488-labeled donkey anti-mouse IgG (H+L) (catalog no. A-21202, Thermo Fisher) at 1:400 dilution. Labeling was performed for 30 min at 4°C in the dark with three washes of FACS buffer performed after each labeling step. Cells were analyzed using a BD LSR II machine (BD Biosciences). Median fluorescence was normalized to unstained control. For *in vivo* work, cells were recovered by BAL fluid as above and were washed twice in FACS buffer prior to addition of fluorophore-conjugated primary antibodies, counting beads (Bangs Laboratories), and viability dye (Live/Dead fixable blue dead cell stain kit; Thermo Fisher). Labeling was performed for 30 min at 4°C in the dark, with three washes of FACS buffer performed after each labeling step. The primary antibodies (plus dye conjugate) used were anti-CD45 (Alexa Fluor 700; BioLegend), anti-Ly6G (BV605; BD Biosciences), anti-CD11b (Alexa Fluor 594; BioLegend), anti-SiglecF (Alexa Fluor 647; BD Biosciences), anti-major histocompatibility complex II (MHC-II) (APC-Cy7; BioLegend), anti-CD31 (FITC; Thermo Fisher), anti-CD36 (PE-Cy7; BioLegend), and anti-EpCAM (BV421; BioLegend). Alveolar macrophages (AM) are defined here as live CD45^+^ cells that are SiglecF^+^ MHC-II^+^, while neutrophils are defined as live CD45^+^ cells that are CD11b^+^ Ly6G^+^ (gating schema in Fig. S6 in the supplemental material). The *t*-distributed stochastic neighbor-embedding algorithm (t-SNE) was implemented in R using package Rtsne, using CD45^+^ 4′,6-diamidino-2-phenylindole negative (DAPI^−^) as input cell population and measured parameters of FSC, SSC, Ly6G, CD11b, SiglecF, MHC-II, CD31, CD36, and EpCAM. All flow cytometry data were analyzed via custom pipeline in R using package flowCore.

10.1128/mBio.02399-20.6 FIG S6 Flow cytometry gating schema. Overview of flow cytometry basic gating strategy for *in vivo* work delineating immune cells (CD45^+^ DAPI^−^), alveolar macrophages (SiglecF^+^ MHC-II^+^), and neutrophils (Ly6G^+^ CD11b^+^ SiglecF^−^ MHC-II^−^). Download FIG S6, PDF file, 0.5 MB. Copyright © 2021 Shah et al. 2021 Shah et al. This content is distributed under the terms of the Creative Commons Attribution 4.0 International license.

### Cytokines.

Cytokine and matrix metallopeptidase levels were measured on cell supernatants and BAL fluid via a multiplex array by a commercial vendor (Eve Technologies).

### Real-time quantitative reverse transcription-quantitative PCR.

Whole RNA was extracted using the EZNA Total RNA kit (Omega Bio-Tek) and quantified using microspectrophotometry (NanoDrop One; Thermo Fisher). cDNA was prepared using the High-Capacity cDNA reverse transcription kit (Applied Biosystems). PCR primers (Table S3) were designed using the UCSC genome browser *in silico* PCR and NCBI Primer-BLAST and manufactured commercially (Eurofins Genomics). Quantitation was performed by the threshold cycle (2^−ΔΔ^*^CT^*) method with expression calculated relative to housekeeping gene control (actin) and untreated condition (PBS).

10.1128/mBio.02399-20.9 TABLE S3 Quantitative RT-PCR primers. Download Table S3, PDF file, 0.06 MB. Copyright © 2021 Shah et al. 2021 Shah et al. This content is distributed under the terms of the Creative Commons Attribution 4.0 International license.

### RNA-seq.

Whole RNA was extracted using EZNA Total RNA kit and checked for purity (RNA integrity number [RIN] > 8) and quantity using an Agilent 2100 Bioanalyzer. Sequencing library preparation and massively parallel sequencing were performed by a commercial vendor (Omega Biosciences), using 150-bp paired-end reads on the Illumina HiSeq 2500 platform. Raw data were processed using a custom pipeline as follows. Quality control performed with FastQC (Babraham Institute) showed that, on average, each run generated >30 Mb of sequence, with a mean base quality score of >38 (i.e., error rate < 0.0002). Sequence reads were analyzed via a custom Python pipeline using existing tools. Reads were first mapped to the human genome (human genome assembly GRCh38) using HISAT2 (80) run with default parameters, with >90% of reads mapping uniquely. Next, StringTie (81) was run with default parameters to assemble transcripts and generate a consensus transcript set, followed by prepDE.py (http://ccb.jhu.edu/software/stringtie/) to generate count expression tables for input into DESeq2 (22) for count-based differential expression analysis. Within DESeq2, modeling was performed using the default analysis function, which models regularized logarithm (rlog)-transformed read counts per gene using a negative binomial distribution with estimated gene-specific dispersion parameters. Two different models of gene expression were employed, one being a categorical model where HZ was binary (present or absent), and the other where expression was modeled using HZ concentration as a continuous variable. Quality control plots, including MA and dispersion plots generated by DESeq2, and QQ plots comparing *P* values obtained with linear versus categorical modeling as well as a comparative Venn diagram, are shown in Fig. S1 and S2. Adjusted *P* values reported here as per the Benjamini-Hochberg method. Heatmaps and Manhattan plots were generated using pheatmap and manhattan packages in R, the former displayed using a false-color scale showing relative expression data per gene (i.e., “row normalized”). Protein-protein interaction networks were generated using StringDB and Cytoscape. A consensus list of top hits was used as input, comprising the union of top 20 hits according to *P*_adj_ for categorical and linear models, with only nodes linked with moderate confidence per StringDB displayed (*n* = 22). Gene set enrichment analyses (GSEA) (39) were performed using R packages fgsea and ReactomePA (40, 41), mapping against curated public data from the Molecular Signatures Database (MSigDB; Broad Institute) including Reactome, Gene Ontology Biologic Process (GO:BP), and Kyoto Encyclopedia of Genes and Genomes (KEGG).

### Data availability.

The data sets supporting the current study were deposited at NCBI GEO (accession no. GSE136007). Analysis scripts are available from the corresponding author on request.
