# Supplementary figures and images for: Hemozoin Promotes Lung Inflammation via Host Epithelial Activation
Source: mBio. 2021 Feb 9;12(1):e02399-20. doi: 10.1128/mBio.02399-20 (PMC7885402; doi:10.1128/mBio.02399-20)

**A**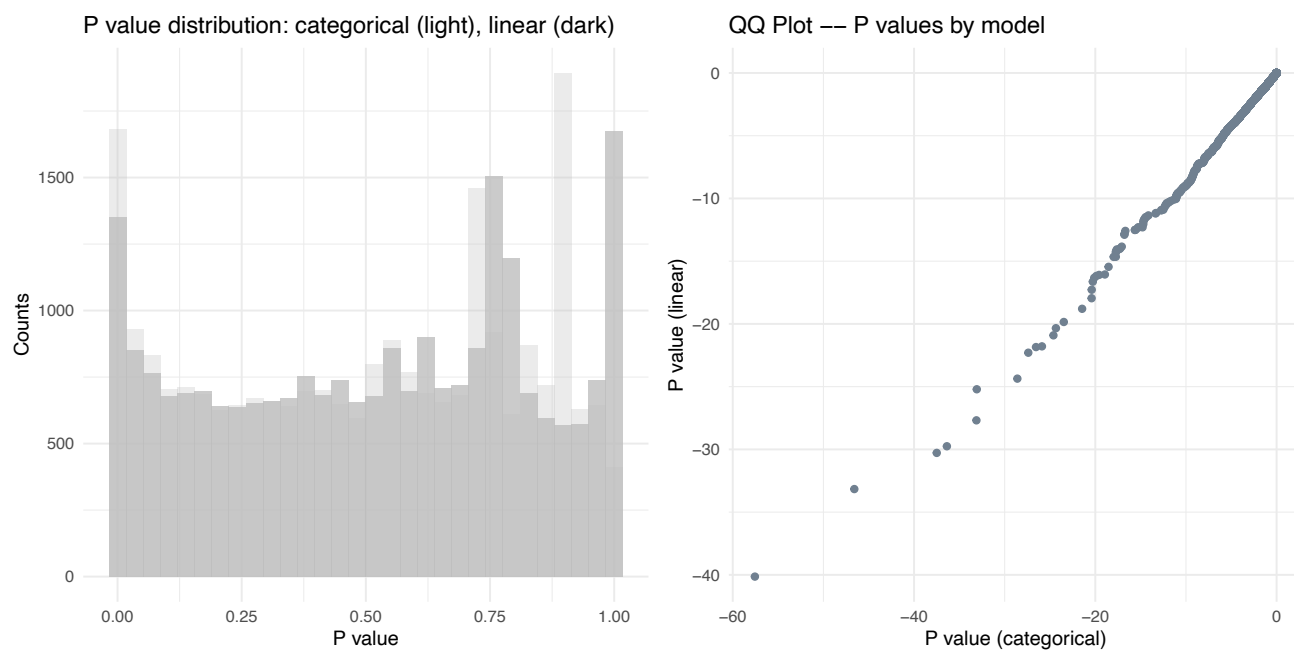**B**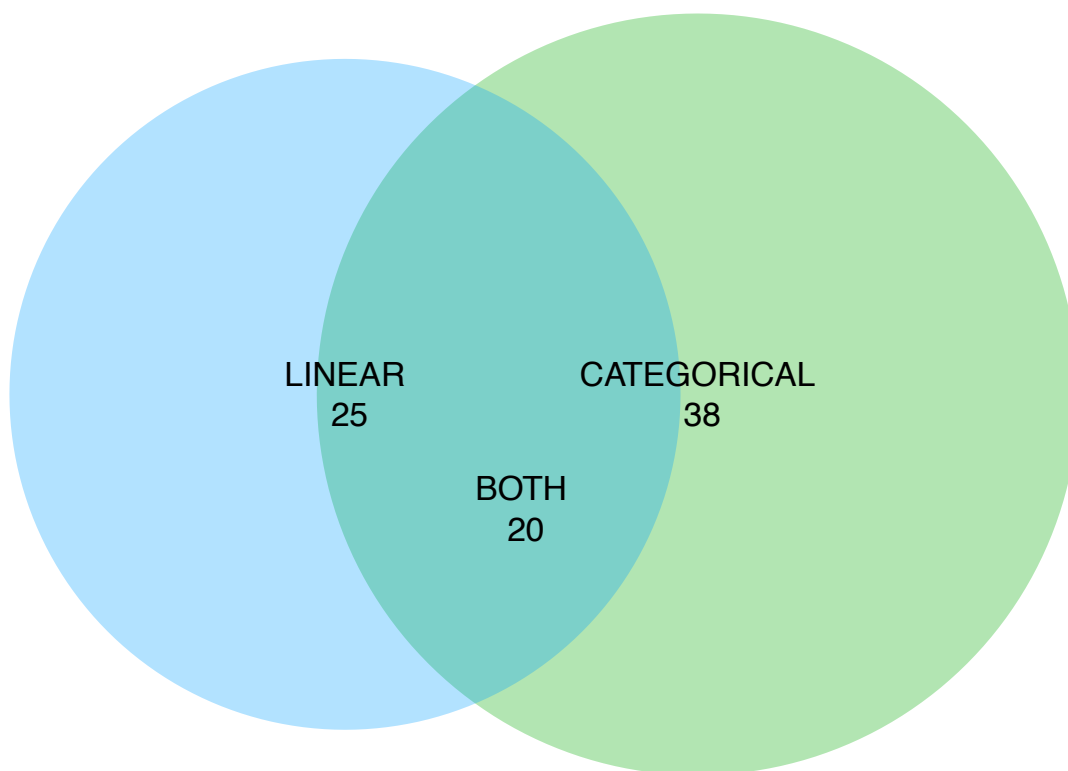

Supplement: FIG S1 [file mBio.02399-20-sf001.pdf]

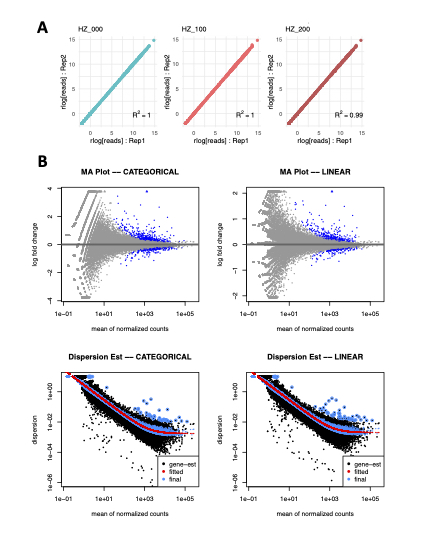

Supplement: FIG S2 [file mBio.02399-20-sf002.jpg]

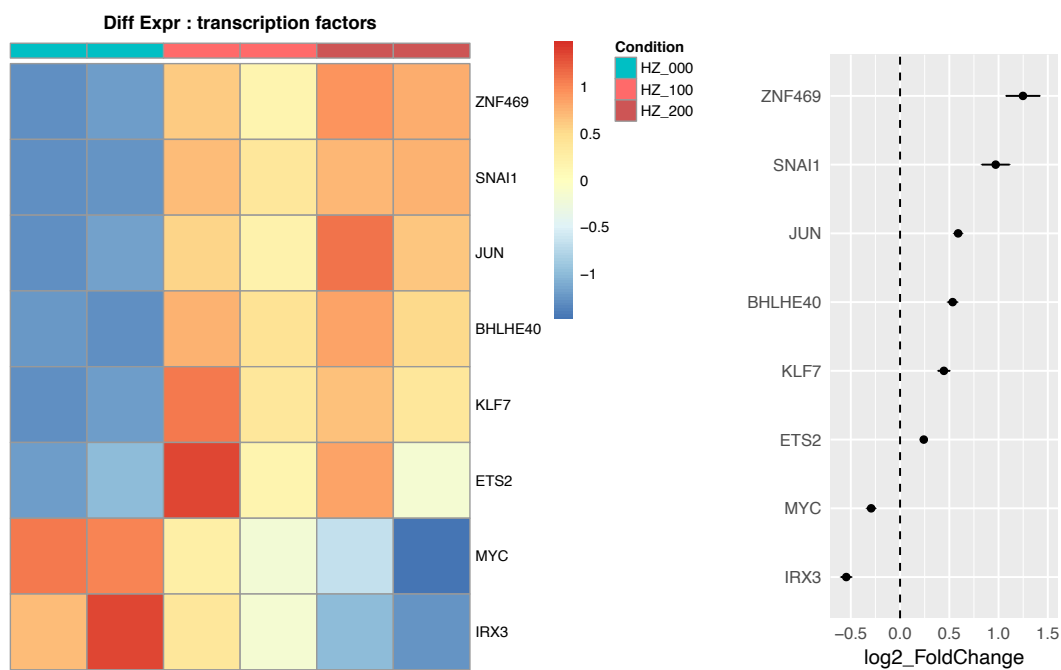

Supplement: FIG S3 [file mBio.02399-20-sf003.pdf]

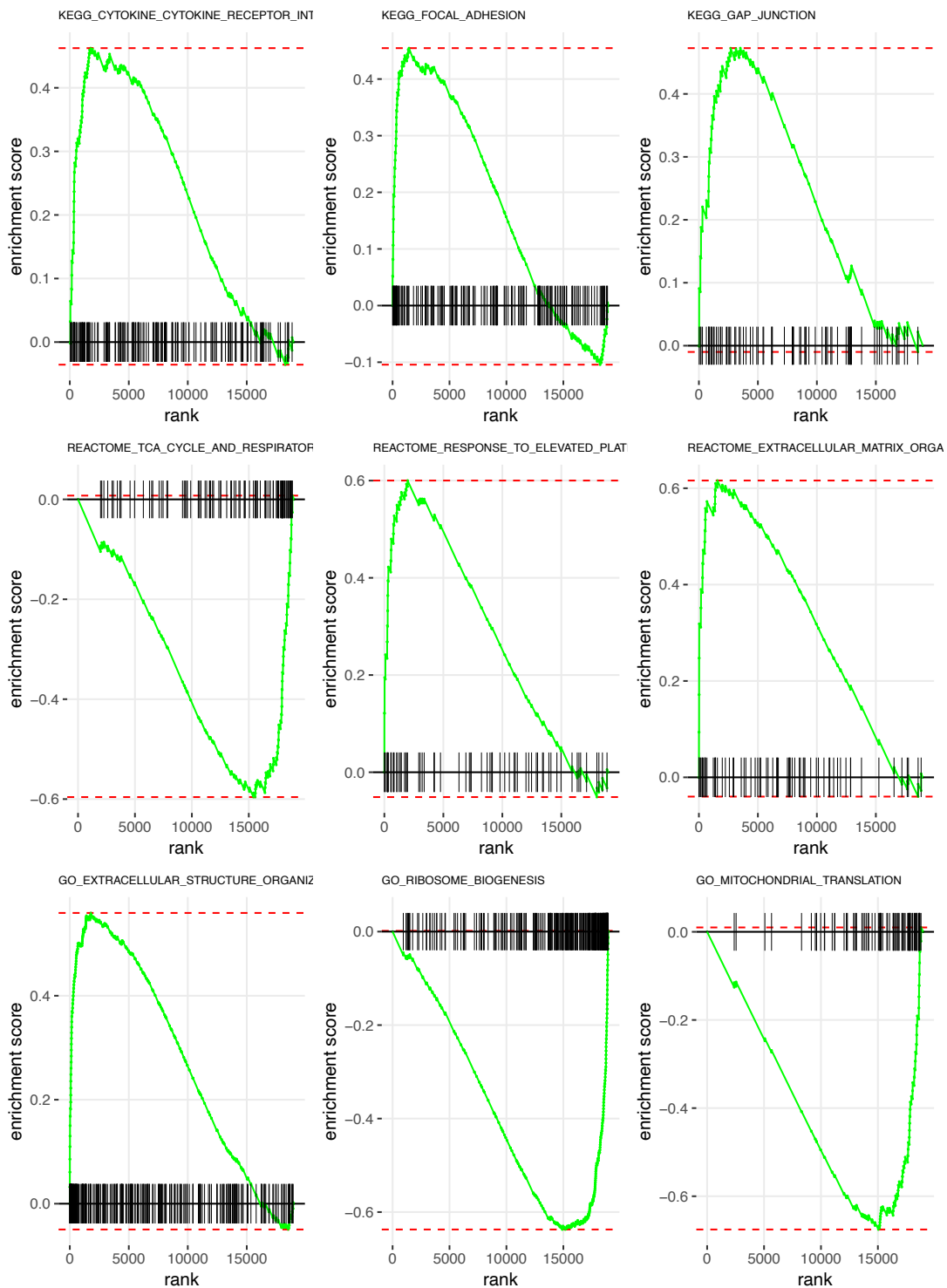

Supplement: FIG S4 [file mBio.02399-20-sf004.pdf]

A

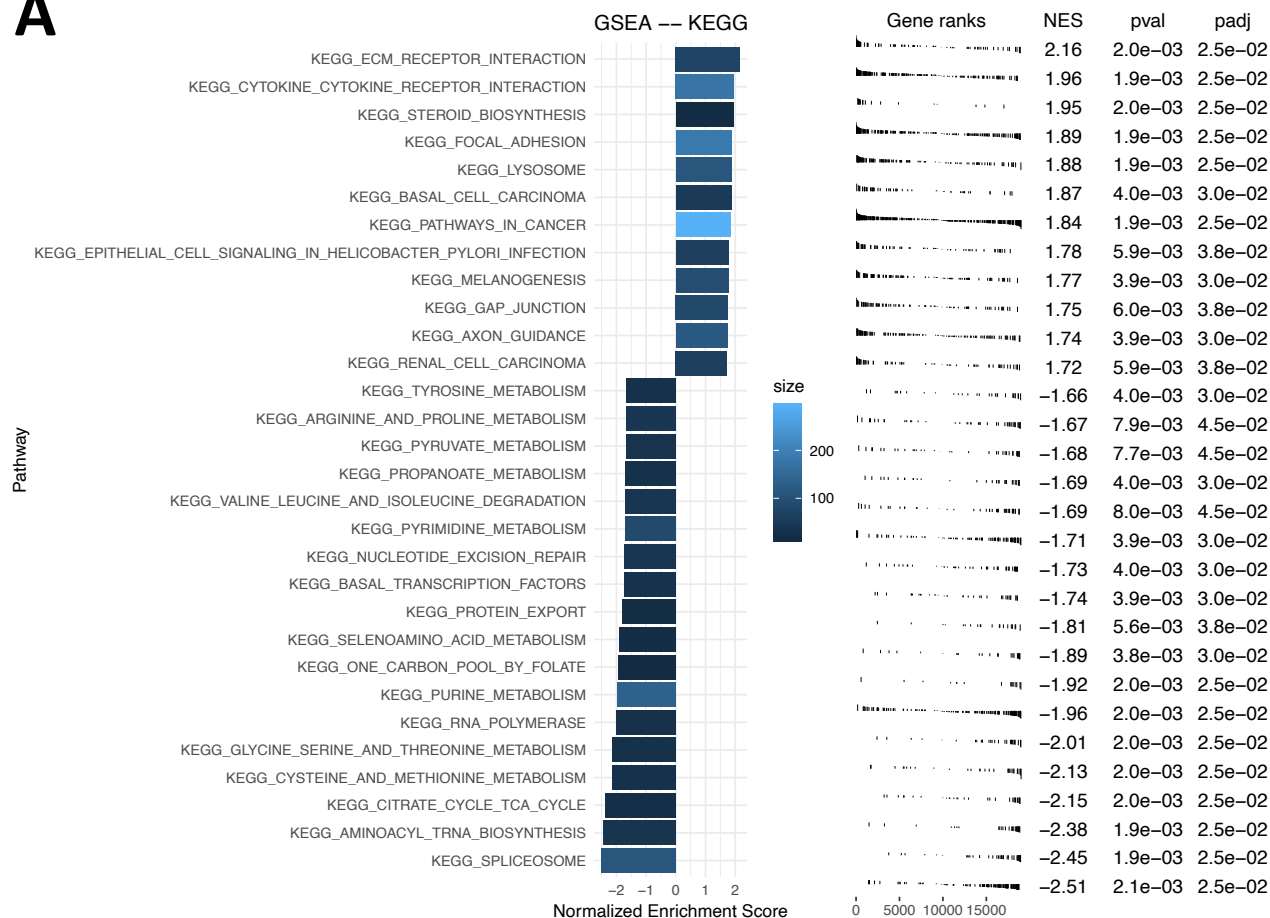

B

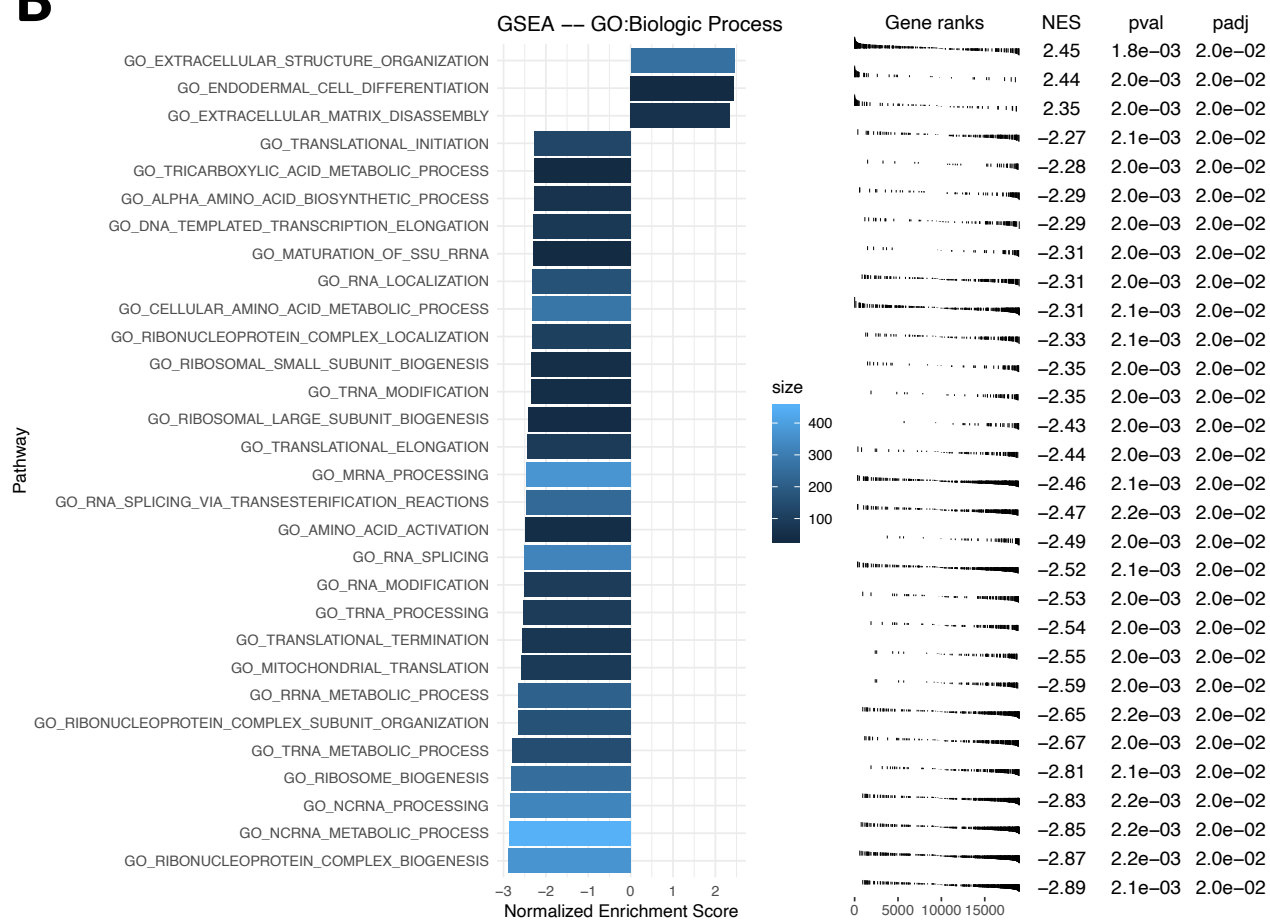

C

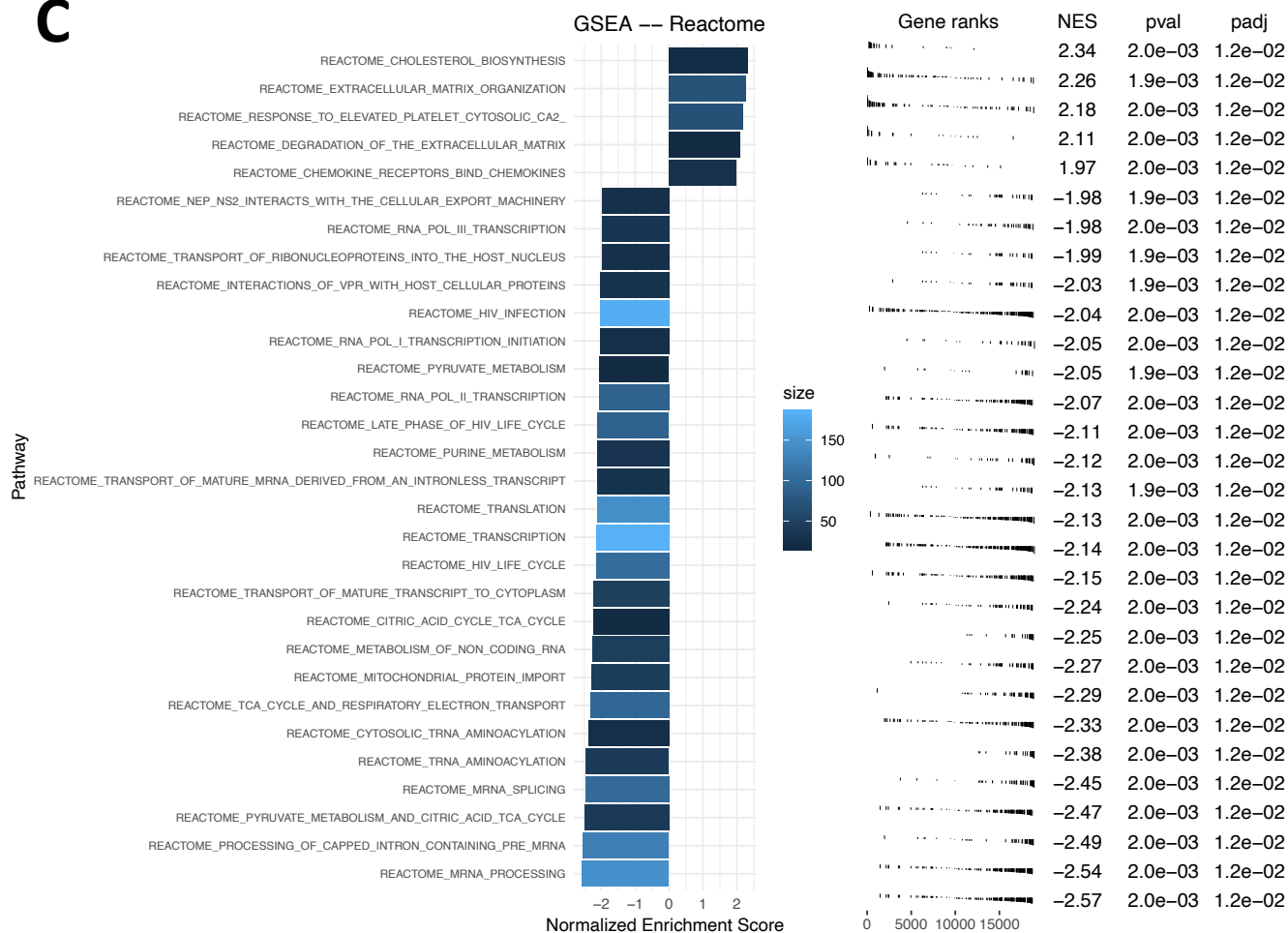

Supplement: FIG S5 [file mBio.02399-20-sf005.pdf]
