# Supplementary material for: Hemozoin Promotes Lung Inflammation via Host Epithelial Activation
Source: mBio. 2021 Feb 9;12(1):e02399-20. doi: 10.1128/mBio.02399-20 (PMC7885402; doi:10.1128/mBio.02399-20)
Supplement: TABLE S1 [file mBio.02399-20-st001.pdf]

Table S1: Top hits (categorical model).

HGNC_Symbol	ENSEMBL_ID	Gene_Type	Chr	Log2_FC	P_adj
NDRG1	ENSG00000104419	protein_coding	8	1.15	3.6×10^{-54}
FN1	ENSG00000115414	protein_coding	2	1.18	1.7×10^{-43}
TGFBI	ENSG00000120708	protein_coding	5	0.92	1.4×10^{-34}
GCLM	ENSG00000023909	protein_coding	1	1.22	1.4×10^{-33}
SQSTM1	ENSG00000161011	protein_coding	5	0.70	1.8×10^{-30}
ALAS1	ENSG00000023330	protein_coding	3	-0.88	1.8×10^{-30}
MMP2	ENSG00000087245	protein_coding	16	1.78	4.6×10^{-26}
LTBP2	ENSG00000119681	protein_coding	14	0.92	6.5×10^{-25}
TNFAIP3	ENSG00000118503	protein_coding	6	0.94	4.1×10^{-24}
ADAM19	ENSG00000135074	protein_coding	5	1.28	1.7×10^{-23}
FTH1	ENSG00000167996	protein_coding	11	0.63	2.8×10^{-22}
CYTH1	ENSG00000108669	protein_coding	17	0.83	4.8×10^{-22}
KANK4	ENSG00000132854	protein_coding	1	2.81	3.3×10^{-21}
BHLHE40	ENSG00000134107	protein_coding	3	0.92	3.2×10^{-19}
STC2	ENSG00000113739	protein_coding	5	-0.74	3.2×10^{-18}
BASP1	ENSG00000176788	protein_coding	5	0.77	3.2×10^{-18}
KIF3C	ENSG00000084731	protein_coding	2	0.71	4.0×10^{-18}
HMOX1	ENSG00000100292	protein_coding	22	4.96	5.4×10^{-18}
MMP9	ENSG00000100985	protein_coding	20	2.03	8.7×10^{-18}
SLC25A37	ENSG00000147454	protein_coding	8	0.61	1.7×10^{-17}
COL16A1	ENSG00000084636	protein_coding	1	1.18	7.0×10^{-17}
AQP3	ENSG00000165272	protein_coding	9	-0.90	1.7×10^{-16}
FAP	ENSG00000078098	protein_coding	2	0.97	5.9×10^{-16}
PPL	ENSG00000118898	protein_coding	16	-0.60	9.5×10^{-16}
PCED1B	ENSG00000179715	protein_coding	12	1.11	9.5×10^{-16}
PLEKHA6	ENSG00000143850	protein_coding	1	-0.58	9.5×10^{-16}
TUBA4A	ENSG00000127824	protein_coding	2	0.84	1.1×10^{-15}
MARCKS	ENSG00000277443	protein_coding	6	0.67	2.1×10^{-15}
MYC	ENSG00000136997	protein_coding	8	-0.53	2.5×10^{-15}
PMEPA1	ENSG00000124225	protein_coding	20	2.65	3.5×10^{-15}
FYB1	ENSG00000082074	protein_coding	5	1.19	6.7×10^{-15}
COL5A1	ENSG00000130635	protein_coding	9	1.72	6.9×10^{-15}
FTL	ENSG00000087086	protein_coding	19	0.66	8.0×10^{-15}
ADGRG6	ENSG00000112414	protein_coding	6	-0.54	8.7×10^{-14}
LPCAT2	ENSG00000087253	protein_coding	16	0.89	9.0×10^{-14}
HDAC9	ENSG00000048052	protein_coding	7	0.94	1.0×10^{-13}
AREG	ENSG00000109321	protein_coding	4	-0.62	1.9×10^{-13}
KLF7	ENSG00000118263	protein_coding	2	0.79	1.9×10^{-13}
NKILA	ENSG00000278709	antisense	20	2.22	5.4×10^{-13}
SNAI1	ENSG00000124216	protein_coding	20	1.83	6.2×10^{-13}
PRSS23	ENSG00000150687	protein_coding	11	0.63	6.2×10^{-13}
FSTL3	ENSG00000070404	protein_coding	19	0.67	7.1×10^{-13}
KLHDC7B	ENSG00000130487	protein_coding	22	0.67	7.8×10^{-13}
FBXO32	ENSG00000156804	protein_coding	8	0.77	9.8×10^{-13}
H19	ENSG00000130600	processed_transcript	11	-0.42	1.2×10^{-12}
ZNF469	ENSG00000225614	protein_coding	16	2.37	2.0×10^{-12}
FHOD3	ENSG00000134775	protein_coding	18	0.57	1.2×10^{-11}
AHNAK2	ENSG00000185567	protein_coding	14	1.27	1.3×10^{-11}
UNKL	ENSG00000059145	protein_coding	16	0.78	4.9×10^{-11}
CSF1R	ENSG00000182578	protein_coding	5	0.83	4.9×10^{-11}
