# Supplementary material for: Hemozoin Promotes Lung Inflammation via Host Epithelial Activation
Source: mBio. 2021 Feb 9;12(1):e02399-20. doi: 10.1128/mBio.02399-20 (PMC7885402; doi:10.1128/mBio.02399-20)
Supplement: TABLE S2 [file mBio.02399-20-st002.pdf]

Table S2: Top hits (linear model).

HGNC_Symbol	ENSEMBL_ID	Gene_Type	Chr	Log2_FC	P_adj
JUN	ENSG00000177606	protein_coding	1	0.97	9.6×10^{-37}
CXCL8	ENSG00000169429	protein_coding	4	1.46	4.6×10^{-30}
ITGB6	ENSG00000115221	protein_coding	2	1.16	2.3×10^{-27}
TUBA4A	ENSG00000127824	protein_coding	2	0.84	5.9×10^{-27}
ADAM19	ENSG00000135074	protein_coding	5	1.28	5.5×10^{-25}
BHLHE40	ENSG00000134107	protein_coding	3	0.92	1.4×10^{-22}
SERPINE1	ENSG00000106366	protein_coding	7	1.93	8.3×10^{-22}
IRX3	ENSG00000177508	protein_coding	16	-0.82	8.4×10^{-20}
MMP9	ENSG00000100985	protein_coding	20	2.03	2.1×10^{-19}
LAMB3	ENSG00000196878	protein_coding	1	0.47	2.2×10^{-19}
TNFAIP3	ENSG00000118503	protein_coding	6	0.94	1.5×10^{-18}
LTBP2	ENSG00000119681	protein_coding	14	0.92	5.0×10^{-18}
LPCAT2	ENSG00000087253	protein_coding	16	0.89	1.5×10^{-17}
FSTL3	ENSG00000070404	protein_coding	19	0.67	1.5×10^{-16}
ID1	ENSG00000125968	protein_coding	20	-0.74	9.8×10^{-16}
AQP3	ENSG00000165272	protein_coding	9	-0.90	4.4×10^{-15}
FRMD6	ENSG00000139926	protein_coding	14	0.63	1.8×10^{-14}
AC007686.4	ENSG00000285966	lincRNA	14	0.91	3.5×10^{-14}
AREG	ENSG00000109321	protein_coding	4	-0.62	4.6×10^{-14}
LAMC2	ENSG00000058085	protein_coding	1	0.56	5.3×10^{-14}
CYTH1	ENSG00000108669	protein_coding	17	0.83	5.5×10^{-14}
NET1	ENSG00000173848	protein_coding	10	0.47	2.1×10^{-13}
SLCO4A1	ENSG00000101187	protein_coding	20	-0.67	1.2×10^{-12}
MPZL2	ENSG00000149573	protein_coding	11	-0.59	1.2×10^{-12}
CHST11	ENSG00000171310	protein_coding	12	0.97	2.7×10^{-12}
PHLDB1	ENSG00000019144	protein_coding	11	0.70	3.2×10^{-12}
PPL	ENSG00000118898	protein_coding	16	-0.60	4.2×10^{-12}
SEMA7A	ENSG00000138623	protein_coding	15	0.72	4.4×10^{-12}
PPP1R3B	ENSG00000173281	protein_coding	8	0.74	4.8×10^{-12}
ETS2	ENSG00000157557	protein_coding	21	0.39	6.4×10^{-12}
NKILA	ENSG00000278709	antisense	20	2.22	5.5×10^{-11}
PTGES	ENSG00000148344	protein_coding	9	-0.40	6.5×10^{-11}
CSF1R	ENSG00000182578	protein_coding	5	0.83	1.0×10^{-10}
IGF1R	ENSG00000140443	protein_coding	15	0.61	1.2×10^{-10}
KLF7	ENSG00000118263	protein_coding	2	0.79	1.2×10^{-10}
UPP1	ENSG00000183696	protein_coding	7	0.58	1.2×10^{-10}
SQSTM1	ENSG00000161011	protein_coding	5	0.70	1.7×10^{-10}
ZNF469	ENSG00000225614	protein_coding	16	2.37	1.7×10^{-10}
PDXK	ENSG00000160209	protein_coding	21	0.41	1.7×10^{-10}
KRT5	ENSG00000186081	protein_coding	12	-0.37	3.0×10^{-10}
DNAJB2	ENSG00000135924	protein_coding	2	0.59	4.7×10^{-10}
CXCL1	ENSG00000163739	protein_coding	4	1.26	6.3×10^{-10}
LAMA3	ENSG00000053747	protein_coding	18	0.44	6.9×10^{-10}
SMAD7	ENSG00000101665	protein_coding	18	1.20	9.3×10^{-10}
SLC25A37	ENSG00000147454	protein_coding	8	0.61	9.9×10^{-10}
HDAC9	ENSG00000048052	protein_coding	7	0.94	1.3×10^{-09}
CYP1B1	ENSG00000138061	protein_coding	2	-0.52	1.8×10^{-09}
SNAI1	ENSG00000124216	protein_coding	20	1.83	1.9×10^{-09}
FAP	ENSG00000078098	protein_coding	2	0.97	3.0×10^{-09}
KLF10	ENSG00000155090	protein_coding	8	0.43	3.2×10^{-09}
