# Supplementary material for: Hemozoin Promotes Lung Inflammation via Host Epithelial Activation
Source: mBio. 2021 Feb 9;12(1):e02399-20. doi: 10.1128/mBio.02399-20 (PMC7885402; doi:10.1128/mBio.02399-20)
Supplement: FIG S6 [file mBio.02399-20-sf006.pdf]

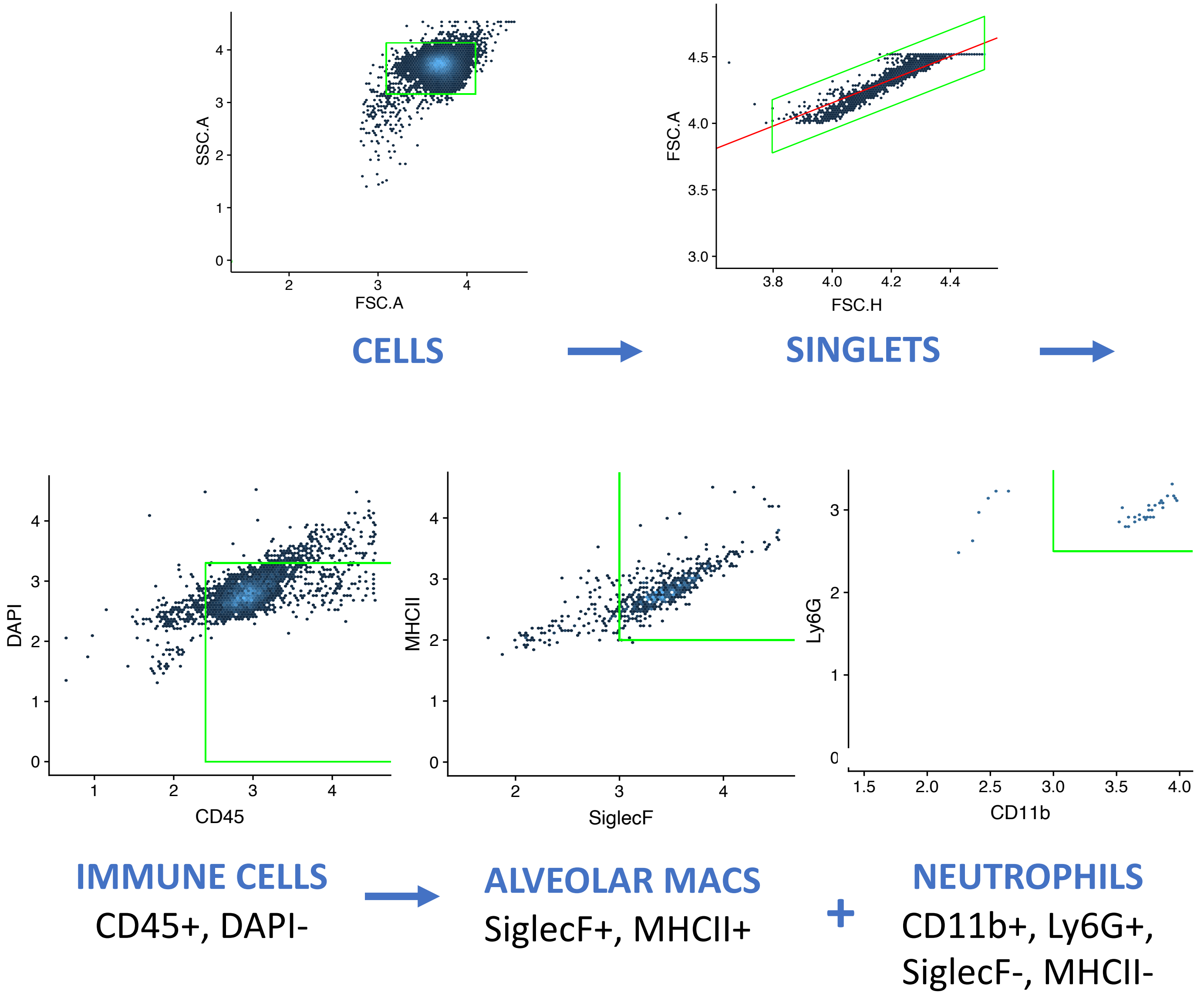
SSC.A
FSC.A
CELLS
FSC.A
FSC.H
SINGLETS
DAPI
CD45
IMMUNE CELLS
CD45+, DAPI-
MHCII
SiglecF
ALVEOLAR MACS
SiglecF+, MHCII+
+
Ly6G
CD11b
NEUTROPHILS
CD11b+, Ly6G+,
SiglecF-, MHCII-
