# Supplementary material for: Hemozoin Promotes Lung Inflammation via Host Epithelial Activation
Source: mBio. 2021 Feb 9;12(1):e02399-20. doi: 10.1128/mBio.02399-20 (PMC7885402; doi:10.1128/mBio.02399-20)
Supplement: TABLE S3 [file mBio.02399-20-st003.pdf]

Table S3: Quantitative RT-PCR primers.

Target	Species	Forward	Reverse
ACTB	human	CATGTACGTTGCTATCCAGGC	CTCCTTAATGTCACGCACGAT
HMOX1	human	AAGACTGCGTTCCTGCTCAAC	AAAGCCCTACAGCAACTGTCG
IL6	human	CTGCAAGAGACTTCCATCCAG	AGTGGTATAGACAGGTCTGTTGG
CD36	human	TTGGGAAAGTCACTGCGACA	AGAGGCAAAGGCCTTGGATG
IL8	human	GTGCAGTTTTGCCAAGGAGT	CTCTGCACCCAGTTTTCCTT
ICAM1	mouse	GTGATGCTCAGGTATCCATCCA	CACAGTTCTCAAAGCACAGCG
